# Myalgic Encephalomyelitis/Chronic Fatigue Syndrome Diagnosis and Management in Young People: A Primer

**DOI:** 10.3389/fped.2017.00121

**Published:** 2017-06-19

**Authors:** Peter C. Rowe, Rosemary A. Underhill, Kenneth J. Friedman, Alan Gurwitt, Marvin S. Medow, Malcolm S. Schwartz, Nigel Speight, Julian M. Stewart, Rosamund Vallings, Katherine S. Rowe

**Affiliations:** ^1^Division of General Pediatrics and Adolescent Medicine, Johns Hopkins University School of Medicine, Baltimore, MD, United States; ^2^Independent Researcher, Palm Coast, FL, United States; ^3^Pharmacology and Physiology, New Jersey Medical School, Newark, NJ, United States; ^4^Yale Child Study Center, Harvard Medical School, University of Connecticut School of Medicine, Newton Highlands, MA, United States; ^5^Division of Pediatric Gastroenterology, Hepatology and Nutrition, New York Medical College, Valhalla, NY, United States; ^6^Drexel University College of Medicine, Philadelphia, PA, United States; ^7^Paediatrician, Durham, United Kingdom; ^8^Division of Pediatric Cardiology, New York Medical College, Valhalla, NY, United States; ^9^Primary Care/Chronic Fatigue Syndrome Clinic, Howick Health and Medical, Auckland, New Zealand; ^10^Department of General Medicine, Royal Children’s Hospital, Murdoch Children’s Research Institute, Melbourne, VIC, Australia

**Keywords:** myalgic encephalomyelitis, chronic fatigue syndrome, pediatric, adolescent, diagnosis, management, postural tachycardia syndrome, joint hypermobility

## Abstract

Myalgic encephalomyelitis/chronic fatigue syndrome (ME/CFS) is a complex disease that affects children and adolescents as well as adults. The etiology has not been established. While many pediatricians and other health-care providers are aware of ME/CFS, they often lack essential knowledge that is necessary for diagnosis and treatment. Many young patients experience symptoms for years before receiving a diagnosis. This primer, written by the International Writing Group for Pediatric ME/CFS, provides information necessary to understand, diagnose, and manage the symptoms of ME/CFS in children and adolescents. ME/CFS is characterized by overwhelming fatigue with a substantial loss of physical and mental stamina. Cardinal features are malaise and a worsening of symptoms following minimal physical or mental exertion. These post-exertional symptoms can persist for hours, days, or weeks and are not relieved by rest or sleep. Other symptoms include cognitive problems, unrefreshing or disturbed sleep, generalized or localized pain, lightheadedness, and additional symptoms in multiple organ systems. While some young patients can attend school, on a full or part-time basis, many others are wheelchair dependent, housebound, or bedbound. Prevalence estimates for pediatric ME/CFS vary from 0.1 to 0.5%. Because there is no diagnostic test for ME/CFS, diagnosis is purely clinical, based on the history and the exclusion of other fatiguing illnesses by physical examination and medical testing. Co-existing medical conditions including orthostatic intolerance (OI) are common. Successful management is based on determining the optimum balance of rest and activity to help prevent post-exertional symptom worsening. Medications are helpful to treat pain, insomnia, OI and other symptoms. The published literature on ME/CFS and specifically that describing the diagnosis and management of pediatric ME/CFS is very limited. Where published studies are lacking, recommendations are based on the clinical observations and practices of the authors.

## Preface

Myalgic encephalomyelitis (ME), also known as chronic fatigue syndrome (CFS) or ME/CFS, affects children and adolescents as well as adults. This primer has been written to provide the information necessary to understand, diagnose, and manage the symptoms of ME/CFS in children and adolescents. Some information in this primer overlaps with information in a similar primer for adults “Chronic Fatigue Syndrome, Myalgic Encephalomyelitis, Primer for Clinical Practitioners” that was published under the sponsorships of the IACFS/ME and is available at www.iacfsme.org. This pediatric primer was developed to cover the many unique aspects of the diagnosis and management of pediatric ME/CFS.

The published literature on pediatric ME/CFS is modest. Many studies on ME/CFS have only enrolled adults. Where it has been helpful, we have sometimes cited studies in adults and this is explained in the text. Where published studies are lacking, our recommendations are based on the clinical expertise of experienced medical practitioners.

The text was developed by consensus of the members of International Writing Group for Pediatric ME/CFS who have made every effort to ensure that the information is accurate and up to date. Statements, opinions, and study results published in this primer are those of the authors and the studies cited. The recommendations contained in any part of this primer do not indicate an exclusive course of treatment or course of action. Nothing contained in this primer should serve as a substitute for the medical judgment of a treating provider.

## Introduction and Overview

Pediatric myalgic encephalomyelitis/chronic fatigue syndrome (ME/CFS) is a complex disease characterized by overwhelming fatigue and a substantial loss of physical and cognitive function. The etiology is uncertain and there is no curative treatment. The cardinal feature is a sensation of feeling ill (malaise) and worsening of symptoms following minimal physical or mental exertion. This post-exertional worsening can persist for hours, days, or weeks, and is not relieved by rest or sleep. Other symptoms include unrefreshing or disturbed sleep, cognitive impairment, and a multitude of immune, neurological, and autonomic symptoms. Orthostatic intolerance (OI) is a common co-morbid condition.

Significant pathophysiological changes found in ME/CFS show that it is an organic/physical illness. Secondary psychological symptoms can be present in some patients as occurs in many other chronic illnesses, but psychological factors have not been shown to be the cause.

### Nomenclature

Myalgic Encephalomyelitis (ME) and chronic fatigue syndrome (CFS) were names given to two well-documented cluster outbreaks of a clinically similar illness in London, UK in 1955 and in NV, USA in 1984. Several different but overlapping case definitions have been published for ME and for CFS to aid in diagnosing sporadic cases. Research studies tend to use the term CFS because a case definition was written for this purpose (1). The name CFS has been criticized for trivializing the illness (2), and it can be confused with the non-specific term chronic fatigue, which is a common symptom in other illnesses. The World Health Organization classifies ME as a disease of the central nervous system, G93.3 (3).

Less common names for the illness include chronic fatigue immune dysfunction syndrome, myalgic encephalopathy and neuro-endocrine-immune dysfunction syndrome. In 2015 a new name, SEID and a new case definition were suggested (2). Currently the new name is under discussion and the new case definition has not yet been clinically validated. This publication will use the acronym ME/CFS.

Symptoms of ME/CFS sometimes follow an acute illness, such as influenza or infectious mononucleosis (4). If symptoms resolve within 6 months, the term post-infectious fatigue syndrome is used to describe the illness.

### Epidemiology

Myalgic encephalomyelitis/chronic fatigue syndrome is globally endemic. Most cases are sporadic, but cluster outbreaks have occurred worldwide. In several outbreaks the illness has been prominent in schoolchildren (5, 6). In sporadic cases the disease is not thought to be transmitted by casual contact. ME/CFS affects all ages, races and socioeconomic groups. In sporadic ME/CFS, two peak ages of onset are seen, 11–19 years in young patients and 30–39 years in adults (7). Although adolescents are more likely than younger children to have ME/CFS, children as young as 2 years old have developed the illness (5, 8). In adolescents, approximately 3–4 times as many girls as boys have ME/CFS. There are less data on the sex ratio in younger children. Estimates of the prevalence of pediatric ME/CFS vary in different studies from 0.1 to 0.5% (9, 10). Research studies have shown that 84–91% of adult patients who satisfy diagnostic criteria for ME/CFS have not been diagnosed (11, 12). We are aware of one comparable study having been done in children and adolescents (13).

### Presentation, Course of the Illness, Prognosis

Myalgic encephalomyelitis/chronic fatigue syndrome can begin suddenly, gradually, or with an abrupt increase in the intensity and frequency of milder chronic symptoms. There can be a history of repeated minor relapsing and remitting prodromal illnesses over the months or years preceding the onset. An acute onset of fever and viral-like symptoms is common, and the onset also can be marked by severe orthostatic symptoms. ME/CFS can follow a known illness such as infectious mononucleosis (4). A gradual onset is more common in younger children and can occur over months or years.

While all patients experience a substantial loss of physical and cognitive functioning, there is a wide spectrum of severity. Mildly affected young people might be able to attend school full-time or part-time, but they might have to limit sport and after-school activities and have frequent school absences. ME/CFS has been found to be the most common cause of long-term absence from school (9, 13–16). More severely affected young people can be wheelchair dependent, housebound, or bedbound. The more impaired might even have difficulty participating in home tutoring sessions. In young persons with ME/CFS, overall self-reported quality of life is often lower than in other illnesses such as diabetes, epilepsy, and cystic fibrosis (17, 18).

Symptoms often fluctuate significantly during the day and from day-to-day. Commonly, patients are slow to get moving upon awakening, with somewhat better function later in the day. Reduced ability to function after activity (physical, cognitive, emotional, orthostatic stress, or academic pressure)—often referred to as “a crash” by patients—with prolonged recovery is a feature. In girls, ME/CFS symptoms are often worse at or just before the menstrual period. The unpredictable level of function from day-to-day can interfere with planning ahead for school attendance, social outings, or family obligations.

The course of ME/CFS is very unpredictable but must often be measured in years, not weeks or months. Remissions and relapses are common. Relapses can be caused by overexertion, infectious illnesses or failure to recover from a “crash” (see above). Dramatic improvement sometimes occurs in the first 4 years, but slow improvement over time is more likely.

It is generally accepted that young people with ME/CFS have a more favorable prognosis than adults. There have been few studies with sufficient numbers and duration of follow-up to be confident of the findings, but factors such as severity of symptoms or age at onset have not been shown to be reliable predictors of long-term outcomes. In a follow-up study of nearly 700 young people the average duration of illness of those who report having “recovered” was 4–5 years with a range from 1 to 15 years. By 5 years, 60% reported recovery, and by 12 years, 88% reported recovery. Of those who reported recovery, about one-third admitted to modifying their activities to remain feeling well (19). Several other studies found that although many patients improved, 20–48% showed no improvement or actually had worse fatigue and physical impairment at follow-up times ranging from 2 to 13 years (20–22). Even among those who report having completely recovered, many describe persistent symptoms that are not reported by healthy individuals (20).

Feedback from young people indicated that an important determinant of their functioning as adults was the effort made to enable them to remain engaged in education. This might have followed relatively unconventional pathways but it enabled them to remain socially connected and to feel they were able to achieve their aspirations fully or in part. From this group, more than 95% were either studying or working part or fulltime (23).

### Diagnosis

No valid, reliable, laboratory test that confirms the diagnosis is currently available. The diagnosis of ME/CFS is purely clinical and is based on the history and the exclusion of other fatiguing illnesses by physical examination and medical testing. Routine blood tests are usually normal. If the typical symptom pattern is not recognized, the diagnosis will be overlooked. The diagnosis depends on the patient’s symptoms meeting the criteria of one of several overlapping case definitions (1, 2, 24–27). Most of the case definitions were developed for adults and they can exclude some young people with ME/CFS. Some case definitions are also quite complex to use in primary care and some do not require the cardinal symptom of post-exertional exacerbation of symptoms to be present. We recommend the diagnostic criteria shown in Section “Clinical Diagnosis.”

### Role of the Health Practitioner in Diagnosis and Management

Young people who appear to have ME/CFS should be evaluated by a physician. A comprehensive history, a thorough physical examination, and appropriate laboratory testing are necessary to make the diagnosis and to exclude other fatiguing illnesses. Co-morbid illnesses are common and require appropriate treatment (see Comorbid Medical Conditions). Some patients with an initial diagnosis of ME/CFS are later found to have a different treatable illness.

Establishing a diagnosis frequently provides the patient and parents much relief. Early diagnosis of ME/CFS can lessen the impact of the illness through timely support and intervention. The unequivocal advice for careful avoidance of overexertion can help to both avoid deterioration and facilitate improvement.

Since there is no medication or intervention which will cure ME/CFS, clinical care focuses on managing symptoms and improving function. A management plan might include:

- Educating the patient, the parents, the family, and the school about the illness (e.g., using handouts, see Appendices C–E).
- Guidance on determining the optimum balance of rest and activity to help prevent post-exertional symptom worsening.
- Advice on diet, social interactions, and education.
- The treatment of symptoms with non-pharmacological interventions and/or medications.
- Regular assessment of progress and watchfulness for the emergence of other illnesses.

The chronicity of ME/CFS signifies the need for continuing management and periodic re-evaluation. Regular monitoring can support the young patient and uncover a change of symptoms, or the emergence of a new illness. Young patients can do well when treated in a primary care setting, but given the complexity of this illness, appropriate referral to other health practitioners (preferably those familiar with ME/CFS) is often needed.

The health practitioner has an important role helping to ensure that the young patient receives the most appropriate schooling by educating the young person’s school personnel about the effect of the illness on scholastic performance, and providing appropriate documentation to education authorities.

## Etiology and Pathophysiology

The underlying etiology of ME/CFS has not been established. Well-documented pathophysiological changes demonstrate that ME/CFS is a multisystem physical disease, not a psychological disorder. The wide variety of pathophysiological findings has led to multiple hypotheses for etiology. These include: infectious agents, immune dysfunction, autoimmune disorders, circulatory abnormalities, neuroendocrine disorders, metabolic disturbances, brain dysfunction, toxins, genetic susceptibility, abnormal gene expression, or a combination of any of these mechanisms.

### Etiological Factors

There is evidence that several predisposing and precipitating factors can contribute to the illness, but evidence for perpetuating factors is limited.

#### Predisposing Factors

Being female is a predisposing factor in post-pubertal adolescents. The prevalence of ME/CFS is 3–4 times higher in adolescent girls than in boys (9, 28). There is less information on the sex ratio in younger children. Genetic factors may produce a susceptibility to the illness in some families. Studies have shown that in approximately 20% of patients, ME/CFS affects more than one family member and in 90% of them, the affected relatives were genetically related (29). The prevalence of ME/CFS was found to be 5.1% in the offspring of mothers with ME/CFS (29). Another study showed an excess relative risk for developing ME/CFS in first (2.7), second (2.3), and third (1.9) degree relatives (30). Twin studies have shown that the concordance rate for a ME/CFS-like illness was 55% in monozygotic twins and 19% in dizygotic twins (31). Approximately 60% of adolescents with ME/CFS have joint hypermobility, compared to approximately 20% of healthy adolescents (32). The mechanism by which this changes the risk of illness is not understood.

#### Precipitating Factors

Sporadic cases of ME/CFS can be preceded (triggered) by: a viral, bacterial, or parasitic infection, an immunization, significant physical or emotional trauma, overexertion, (“overtraining”) chronic sleep deprivation, exposure to a toxin, or an atypical adverse reaction to a medication. In some patients no precipitating factor can be identified.

#### Perpetuating Factors

It is difficult to determine factors that perpetuate the illness, although it has been suggested that factors that aggravate the illness can also contribute to its persistence. Few studies have investigated this issue. Aggravating factors include: failure to diagnose the illness promptly, resulting in poor management in the early stages of the illness, overexertion resulting in “crashes,” stress, inadequate sleep, and co-morbid conditions, such as OI.

### Pathophysiological Basis for Symptoms

Despite the wide variability in precipitating factors and in pathophysiological findings, there appear to be some common underlying mechanisms behind the most prevalent symptoms.

#### Infection

Although sporadic cases are more common, ME/CFS can also occur in cluster outbreaks, suggesting an important etiologic role for microbial pathogens. This assumption is supported by the common pattern of an abrupt onset in association with flu-like symptoms in many sporadic cases. In the absence of evidence of persistent replication of an infectious agent, the main scientific debate currently centers around whether there is an occult active persistent infection or whether infectious agents have been cleared, but have triggered chronic symptoms due to a maladaptive host immunologic response.

Pediatric ME/CFS sometimes follows an acute infection, including most prominently Epstein-Barr virus (EBV). After monospot-positive infectious mononucleosis, 13%, 7%, and 4% of adolescents met criteria for ME/CFS at 6, 12, and 24 months after infection, respectively (4). As in adults, the most important factor associated with developing ME/CFS was the severity of the initial illness, as reflected by the number of days spent in bed with acute symptoms (33). Several other ubiquitous infectious agents or their antibodies have been found in patients with ME/CFS and their presence can influence symptom severity. Although individual cases of ME/CFS have been identified in association with B. *burgdorferi*, cytomegalovirus, human herpesvirus 6, Coxsackie virus, enterovirus, adenovirus, or parvovirus B19, a large Norwegian pediatric study did not identify a prominent etiologic role for any of these organisms (34).

Some studies in adult patients with ME/CFS report benefit from anti-viral treatment, suggestive of a viral pathophysiology, but other studies have not confirmed the efficacy of anti-viral treatment (35–37). We are not aware of studies of anti-viral agents in young patients.

#### Immune Dysfunction

Another prominent theory is that ME/CFS symptoms can be a consequence of a prolonged immunologic host response to infection. A limited number of immunological studies have been performed in pediatric patients. Studies in adults show immune system changes that are often inconsistent and tend to wax and wane over time (38). The most consistent immune responses are: immune activation, defective cell-mediated immunity, decreased natural killer cell (NK) cytotoxic activity that correlates with the severity of the illness (39, 40) and the occasional finding of low levels of autoantibodies including rheumatoid factor, anti-thyroid antibodies, anti-gliadin, anti-smooth muscle antibodies, and cold agglutinins in some patients. These immune responses are not unique to ME/CFS.

In pediatric patients, evidence of poor NK cell function is less robust than in adults (41), but few studies have been performed. Individual studies have reported cutaneous anergy (42), increased prevalence of autoantibodies (41), a beneficial response to intravenous immunoglobulin (IVIG) (42), increased rates of apoptosis in peripheral white blood cells (43), and abnormal T-cell inhibitory or proliferative responses to stimuli (44). Although one small study showed elevations or reductions in some cytokine populations (45), a larger investigation found no evidence of cytokine abnormalities (46). Very little research has been devoted as to whether allergies, food intolerance, or mast cell activation play a contributing role in the pathophysiology of ME/CFS symptoms.

A small randomized trial in adults demonstrated improvement in ME/CFS symptoms after rituximab-mediated B-cell depletion (47), but there have been no pediatric studies.

#### Circulatory Abnormalities

Lightheadedness is very common in pediatric ME/CFS, and prolonged upright posture can aggravate other symptoms, including fatigue, headache, nausea, and cognitive dysfunction (48–50). Controlled studies have shown a higher prevalence of postural tachycardia syndrome (POTS) and neurally mediated hypotension (NMH) in pediatric patients with ME/CFS (49, 51–55). More work is needed to define the causes of NMH and POTS. Both can follow infectious illnesses, and themselves can be secondary to autoimmune phenomena. Other circulatory abnormalities in pediatric ME/CFS include a delayed recovery of cerebral oxygenation after a brief period of standing compared to healthy controls (54), and the demonstration that cognitive problems are exacerbated by orthostatic stress (50, 56).

Low blood volume has also been found in some adult patients with ME/CFS (57–60). The initiation of treatments directed at OI can sometimes relieve ME/CFS symptoms in pediatric as well as adult patients (see Orthostatic Intolerance).

#### Neuroendocrine Abnormalities

The similarity in symptoms between ME/CFS and adrenal insufficiency has prompted investigation into abnormal hypothalamic-pituitary-adrenal axis function in both adults and adolescents. Several (albeit not all) pediatric ME/CFS studies have identified statistically lower cortisol levels and urine cortisol:creatinine values in ME/CFS patients compared to healthy controls (34, 44, 61–63). However, the cortisol values in those with ME/CFS are still within the normal range, raising questions regarding the clinical relevance of these findings. Treating ME/CFS with supplemental cortisol brings only modest clinical benefits, and in adult studies is associated with the development of potentially life-threatening adrenal insufficiency (64).

#### Brain Imaging Abnormalities

The high prevalence of cognitive dysfunction has focused attention on the hypothesis that abnormalities in the central nervous system are important in the pathophysiology of ME/CFS. Using several brain imaging techniques, some small studies in adults have shown a variety of differences between ME/CFS patients and controls including increased activation of microglia or astrocytes (65), volume loss in white and gray matter (66, 67), reductions in absolute cerebral blood flow (68), and increased ventricular lactate (69, 70). Both pediatric and adult studies have shown that patients activate a wider distribution of neural resources to perform a specific cognitive task (71–73). Improving brain blood flow improves cognitive performance in those with ME/CFS (74).

#### Metabolic Abnormalities

Profound exhaustion and post-exertional worsening of symptoms are hallmarks of ME/CFS. Adult studies have shown impaired oxygen consumption during exercise and activation of anaerobic metabolic pathways in the early stages of exercise (75, 76). When exercise testing is conducted on two consecutive days, there is a decline in exercise performance and an abnormal recovery response (decline in VO2 max) on the second day (77, 78). In single day pediatric exercise studies, those with CFS exercised less efficiently than controls who had recovered from mononucleosis, but significant differences in peak work capacity were not found (79).

These findings in adults, together with increased ventricular lactate have led to the hypothesis that ME/CFS symptoms might be due to a problem in mitochondrial bioenergetics and ATP production (80, 81). Thus far, the exact role of mitochondrial dysfunction in ME/CFS has not been clearly established.

#### Gene Studies

Studies in adult patients with ME/CFS have shown alterations in the expression of genes controlling immune modulation, oxidative stress and apoptosis. Several subtypes were reported, and the presence of some of these subtypes, correlated with symptom severity (82, 83). Moderate exercise increased the expression of sensory, adrenergic and immune genes in patients with ME/CFS, but not in controls (83). Epigenetic differences between those with ME/CFS and healthy controls have recently been described (84).

The prevailing theories related to pathophysiological mechanisms are not necessarily mutually exclusive. Circulatory dysfunction, for example, can be caused by infections and immune mechanisms, and in turn can have effects on inflammation, oxygen delivery to cells, and microglial activation. A more complete discussion of the potential pathophysiological mechanisms of ME/CFS symptoms is beyond the scope of this primer.

## Adolescent Development

Puberty is defined as the time when a young person’s body, feelings, and relationships change from those of a child into those of an adult. Changes occurring in the young person’s body include a growth spurt, enlargement of the genitalia, development of androgen hair, and the onset of sexual functions including ejaculation or menses. Striking emotional and psychological changes occur. Puberty is a time of significant development of self-awareness, abstract thinking, increased sensitivity, and mood changes.

Myalgic encephalomyelitis/chronic fatigue syndrome preceding puberty can impact the development of both the physical, pubertal changes, and the young person’s emotional state and self-image. There can be delay or acceleration of pubertal changes or alteration of physiological processes including hypo-function or, less commonly, hyper-function of the neuroendocrine system. The development of ME/CFS following puberty can be associated with a delay in normal, psychological development fostered by social isolation. Cessation of menstruation can also occur and this can be of great concern to the patient.

Puberty, with its physiological changes, can also significantly exacerbate pre-existing ME/CFS symptoms. The risk of developing ME/CFS increases after puberty especially in females compared to males. The post-pubertal F:M sex ratio is 3–4:1.

## Clinical Diagnosis

The diagnosis of ME/CFS is based on a careful clinical history, recognition of the pattern of symptoms, and the exclusion of other illnesses that might explain the symptom complex. Currently, there is no valid, reliable laboratory test that confirms the diagnosis. Moreover, although the physical examination is not entirely normal, there are no specific diagnostic clinical signs and frequently the patient does not look ill. The diagnosis is often overlooked or delayed and is sometimes made retrospectively when the child is older.

Several varied clinical criteria are currently used to diagnose ME/CFS in children and adolescents. None have been clinically validated in formal studies. The 1994 Fukuda case definition (1), see Appendix A, is often used, but was designed for research purposes in adults and can exclude some young patients with ME/CFS and include others, who are later found to have another illness (27). The 2003 Canadian clinical case definition (CCC) (24) is widely used in adults, because it emphasizes the core symptoms of the illness. A pediatric case definition based on the CCC was published in 2006 (27). The latter is somewhat complex for use in clinical practice. The following diagnostic criteria are offered by our experienced clinicians and are based on their collective experience and insight. The criteria provide useful diagnostic sensitivity within a heterogeneous pediatric ME/CFS patient population. A symptom severity scoring system is included to increase the specificity of the diagnostic criteria.

Myalgic encephalomyelitis/chronic fatigue syndrome is characterized by numerous symptoms in multiple body systems, but its diagnosis requires only the presence of a group of specific, core symptoms.

- The cardinal feature of ME/CFS is malaise and the exacerbation of symptoms after a variety of forms of effort (most commonly physical or cognitive activity or orthostatic stress). Post-exertional symptoms can persist for hours, days, or weeks and are not relieved by rest. This symptom is uncommon in other illnesses.
- Other core symptoms are impaired physical and/or cognitive function, fatigue, sleep disturbance, cognitive symptoms, and pain.
- Some or all symptoms are present every day (symptoms often fluctuate significantly in intensity during the day or from day-to-day).
- Symptoms are mostly moderate to severe.
- Symptoms have persisted or recurred for at least 6 months (a provisional diagnosis and appropriate management can be instituted before 6 months).
- Other fatiguing illnesses must be excluded by history, physical examination, and medical testing.

The diagnostic criteria are set out as a *Clinical Diagnostic Worksheet* which can be used in clinical practice. The worksheet can also be completed at follow-up visits to confirm the diagnosis, or track progress. Additional symptoms can be present in multiple organ systems. Those that are commoner in young patients include (a) orthostatic intolerance (OI): prolonged upright posture can induce symptoms such as lightheadedness, increased fatigue, cognitive worsening, headaches, and/or nausea. Postural tachycardia syndrome (POTS) or NMH is often present, (b) hypersensitivities to light, noise, touch, odors. and/or medications, (c) thermo-regulatory imbalance including low body temperature, intolerance to heat and cold, and cold hands and feet, (d) gastrointestinal symptoms such as abdominal pain, nausea, and anorexia, (e) worsening of fatigue in the days before and during the onset of menses can occur in young women, and (f) other co-morbid conditions can be present and are discussed in detail in Section “Comorbid Medical Conditions.”

Pediatric ME/CFS: Clinical Diagnostic Worksheet: Patient Name _____________________________________________________ Patient ID ________________________ Date __________

Criteria for the diagnosis of ME/CFS in children and adolescents:

- Impaired function, post-exertional symptoms, fatigue, sleep disturbance, neurocognitive problems, and pain.
- Some or all symptoms are present every day (symptoms often fluctuate significantly in intensity during the day or from day-to-day).
- The symptoms are mostly moderate to severe.
- Symptoms have persisted or recurred for at least 6 months (a provisional diagnosis and appropriate management can be instituted before 6 months).
- Other fatiguing illnesses have been excluded by history, physical examination, and medical testing

**Table:** 

Have the following symptoms persisted or recurred during the past 6 months?	Present	Symptom severity in the past month: absent (0), mild (1), moderate (2), and severe (3)
1. *Impaired function*: there is loss of mental and/or physical stamina and a substantial reduction in ability to take part in personal, educational, and/or social activities	Yes [ ] No [ ]	

2. *Post-exertional symptoms*: normal activity or mild/moderate exertion is followed by worsening of malaise, fatigue, and other symptoms. Recovery takes more than 24 h	Yes [ ] No [ ]	

3. *Fatigue*: the fatigue is not the result of ongoing exertion, is not relieved by rest, and is medically unexplained. Fatigue can worsen with prolonged upright posture	Yes [ ] No [ ]	

4. *Sleep problems*: sleep is unrefreshing with disturbed quantity or rhythm that can include daytime hypersomnia, nighttime insomnia, and day/night reversal	Yes [ ] No [ ]	

5. *Cognitive problems*: any of the following: difficulty in concentration or focusing, difficulty understanding information and/or expressing thoughts, difficulty finding words or numbers, impaired short-term memory, absent mindedness, slowness of thought. Cognitive problems can be provoked by, or worsen with prolonged upright posture and/or physical or mental activity. Some young patients may not recognize these problems, but they might be noticed by a parent or teacher.	Yes [ ] No [ ]	

6. *Pain*: can be widespread or localized, commonly seen are: chronic daily headaches, myalgias, abdominal pain, joint pains, sore throats, and painful lymph nodes. Pain can be worsened by prolonged upright posture. Rarely is pain absent	Yes [ ] No [ ]	

*Total symptom severity score*:		

**Other symptoms present in many, but not all, pediatric patients with ME/CFS:**

*Orthostatic intolerance*: prolonged upright posture can induce symptoms that can include lightheadedness, increased fatigue, cognitive worsening, headaches, and/or nausea. Postural tachycardia syndrome (POTS) or neurally mediated hypotension (NMH) are often present.
*Hypersensitivities*: to light, noise, touch, odors, and medications.
*Thermo-regulatory imbalance*: low body temperature, intolerance to heat and cold, and/or cold hands and feet.
*Gastrointestinal symptoms*: abdominal pain, nausea and/or anorexia.

**To diagnose ME/CFS:**

- Symptom criteria 1, 2, and 3 are present together with at least two of criteria 4, 5, and 6: Yes [ ] No [ ]
- Symptoms are present for 6 months and some or all symptoms are present daily: Yes [ ] No [ ]
- No other diagnosis found from the history, physical examination, and medical testing: Yes [ ] No [ ]
- Symptom severity score: 0–4 ME/CFS unlikely; 5–12 mild/moderate ME/CFS; 13–18 moderate/severe ME/CFS

_______ Patient meets criteria for ME/CFS.

_______ Full criteria not met. Patient should be monitored and symptoms should be managed.

The pattern of ME/CFS symptoms is distinctive and the diagnosis can easily be made in most cases, but there can be some diagnostic challenges. Younger children, especially those under 10 years of age, might not report symptoms accurately. They might not remember having experienced full health and might assume tiredness is normal. The first sign of the illness might be the child’s marked limitation of physical and/or mental activity, noticed by a parent or a teacher. Young patients with ME/CFS might not necessarily recognize that they have symptoms such as cognitive problems or malaise until their health has improved. Some patients might not consider themselves as having post-exertional symptoms because they have learned to pace their activities. Symptoms of ME/CFS wax and wane over time. Some patients might report that one particular symptom was present previously, but has improved by the clinic visit. Symptoms that persist are more easily recalled.

### Patient History

It is critical to allocate enough time for a careful, comprehensive history to be taken from the patient and the parents. The history is not only vital to diagnosis, but also taking the history in a thorough and empathetic manner engenders the trust of the patient and family, shows that the clinician takes the patient’s illness seriously, and is an important prelude to management. Teenagers usually need an opportunity for discussion without a parent present. The initial evaluation might require more than one office visit because the history is sometimes lengthy and the patient might lack sufficient stamina. Some patients might need to lie down during office visits. For other seriously ill patients, an office visit might be impossible, and they require home visits.

The clinician might find it helpful to first ask the patient to list current symptoms in order of severity, to get a sense of the areas that will need to be explored. It is important to ask the patient and the parents what are their major concerns. This can be an opportunity to correct misinterpretations, e.g., “I think I might have cancer.” Then the history requires attention to the full range of defining symptoms of ME/CFS, recording both frequency and severity.

Information about diet, sleep patterns, depressed mood, anxiety, school performance, relationships with family and friends, drug use, and family and developmental history needs to be obtained. The family history might reveal other family members with ME/CFS symptoms and there is also a higher prevalence of disorders such as fibromyalgia, joint hypermobility, temporomandibular joint dysfunction, anxiety, syncope, and irritable bowel disease.

To assess function, ask about what happens when the young person tries to do activities that she/he previously tolerated, and about activities the individual must now limit or avoid. This can indicate very significant life changes and losses experienced by the patient.

Frequent monitoring of the patient once every 1–3 months, depending on the level of illness severity is important. Young patients who are ill appreciate continuing care. Functional improvement can be judged by determining how much activity generates post-exertional worsening of symptoms. Progress should be measured over time, rather than at a single clinic visit. In patients who are improving, fatigue is often the last symptom to resolve, since young patients often prefer to increase their activities and tolerate the resulting fatigue. The practitioner must be alert for the emergence of new symptoms. They might not be related to ME/CFS, but due to another illness.

### Physical Examination

A thorough physical examination, including a neurological exam is important to exclude other causes of fatigue. Physical signs in ME/CFS are subtle and none are diagnostic. Many patients do not look ill, but noticeable facial pallor is sometimes apparent and often precedes the onset of extreme tiredness in the patient. The pallor can be associated with sub-orbital dark shadows. The pharynx can show non-exudative pharyngitis and cervical and axillary lymph nodes might be palpable and tender. Muscle tenderness is a feature of co-morbid juvenile fibromyalgia. Fibromyalgia is less common in children with ME/CFS than in adults. The hands and feet can be unusually cold and dependent rubor of the legs is often present when standing or sitting. The physical exam should include an assessment for common co-morbid conditions such as OI [heart rate (HR) and blood pressure (BP) sitting and standing], joint hypermobility, and postural dysfunctions (see Comorbid Medical Conditions).

At illness onset, pyrexia can be present and this can persist for some weeks. If fever is present several months into the illness, other causes of fever should be sought. In established cases, slightly subnormal temperature, 96.8–98.0°F (36.0–36.7°C), is common. Some children with ME/CFS have higher resting HRs than expected and occasional patients have resting hypotension. More commonly, HR and BP abnormalities emerge in response to upright posture. The diagnosis and management of orthostatic problems is discussed in the section “Comorbid Medical Conditions.”

### Laboratory Testing

Basic laboratory studies (Table 1) should be performed to identify other causes of fatigue and any organ system dysfunction. Most routine laboratory tests are within the normal range, and do not correlate with overall function, even in the presence of substantial debilitation. Measures of iron stores, vitamin B12 level, and screening for celiac disease are recommended because of their subtle or absent manifestations on physical examination.

**Table 1:** Investigation of ME/CFS: routine laboratory testing.

Complete blood count with differential Erythrocyte sedimentation rate Electrolytes Calcium Phosphate Fasting glucose C-reactive protein Liver function: bilirubin, alkaline phosphatase, gamma glutamyl transaminase, alanine transaminase, aspartate transaminase Total protein, albumin/globulin ratio	Renal function: urea, creatinine, glomerular filtration rate Thyroid function: thyroid stimulating hormone, free thyroxine (free T4) Anti-nuclear antibodies Iron studies: serum iron and transferrin or ferritin Vitamin B 12 and folate Vitamin D3, 25-hydroxy cholecalciferol Celiac screening: tissue transglutaminase IgA and IgG, with serum IgA Urinalysis

Additional testing (Table 2) is based on history, examination and laboratory findings. For example, a search for infections such as Lyme disease depends on the symptoms and on whether the young patient resides in, or has traveled to, endemic areas. Serology for some infectious illnesses can be weakly positive. Although standard serology for EBV and CMV can help with categorizing whether the illness might have been initiated by these organisms, these test results usually do not change management. MRIs of the brain and spine are not routinely indicated, but clearly are important if there are abnormal neurological signs.

**Table 2:** Investigation of ME/CFS: tests to be considered when indicated.

Allergic: skin tests, RAST tests, serum tryptase Cardiac: chest x-ray, EKG (ECG), echocardiogram in those with heart murmurs, tilt testing for those with prominent orthostatic symptoms but no hemodynamic abnormalities in the office 10-min standing test Endocrine/metabolic: morning cortisol, FSH, LH, estradiol, testosterone, prolactin, lactate, pyruvate, IGF-1 and IGF-3, thyroid autoantibodies Gastrointestinal: upper GI endoscopy, colonoscopy, gastric emptying study, HIDA scan, ultrasound of gallbladder Gynecological: ultrasound, laparoscopy, ovarian venogram (to evaluate for ovarian varices) Immunological: anti-double stranded DNA, anti-neutrophil cytoplasmic antibodies, quantitative immunoglobulins, functional antibodies, response to pneumococcal vaccination Infectious diseases: infectious mononucleosis, lyme, and other tick-borne diseases (*Babesia microti, Anaplasma phagocytophilum, Ehrlichia chaffeensis*) in endemic areas Neurological: MRI studies if Chiari malformation, cervical stenosis, tethered spinal cord, or MS are suspected Pulmonary: pulmonary function tests Sleep: polysomnography, multiple sleep latency testing Urological: cystoscopy

### Differential Diagnosis, Exclusionary Medical Conditions

Myalgic encephalomyelitis/chronic fatigue syndrome should not be diagnosed if the patient has an identifiable medical or primary psychiatric condition that could plausibly account for the presenting symptoms. If ME/CFS symptoms persist after adequate treatment of other confounding illnesses, a diagnosis of ME/CFS can be considered.

Fatigue is an early symptom in many medical conditions and can be present before the appearance of the diagnostic features of the underlying illness. Careful follow-up over time is needed in order to identify illnesses that might mimic ME/CFS in their early stages. The presence of post-exertional malaise and exacerbation of symptoms after increased cognitive or physical effort increases the likelihood that ME/CFS is the correct diagnosis. The more common conditions in the differential diagnosis are shown in Table 3. For a more comprehensive list of the less common disorders in the differential diagnosis, see Appendix B. If there is diagnostic uncertainty and referral is necessary, it should be preferably to a specialist familiar with ME/CFS.

**Table 3:** Common conditions in the differential diagnosis of ME/CFS.

Adrenal insufficiency Athletic overtraining syndrome Bowel disorders: celiac disease, inflammatory bowel disease, and eosinophilic gastroenteritis Chiari malformation or cervical spine stenosis Lyme disease and other tick-borne infections Major depression	Narcolepsy Obstructive or central apnea Post-concussion syndrome Severe anemias Systemic lupus erythematosis and similar autoimmune conditions Untreated hypo- or hyper-thyroidism

### Co-Existing Medical Conditions

A number of non-exclusionary conditions can co-exist with ME/CFS (Table 4). If present, co-existing conditions should be evaluated independently and treated appropriately. Some co-existing medical conditions that are seen significantly often in young patients with ME/CFS and significantly reduce the patient’s functional capacity are discussed in more detail in the section “Comorbid Medical Conditions.”

**Table 4:** Non-exclusionary overlapping conditions.

*Autoimmune* Sicca syndrome *Cardiovascular* Autonomic dysfunction Mitral valve prolapse Orthostatic intolerance Neurally mediated hypotension Postural tachycardia syndrome (POTS) Delayed orthostatic hypotension or tachycardia *Endocrine/metabolic* Insulin resistance Hypoglycemia Metabolic syndrome Polycystic ovary syndrome Severe obesity	*Gastrointestinal* Eosinophilic esophagitis Gastroesophageal reflux GI motility disturbances IgE-mediated food allergy, non-IgE mediated milk protein or other food protein hypersensitivity Irritable bowel syndrome Lactose/fructose intolerance *Gynecological* Dysmenorrhea Endometriosis Pelvic congestion syndrome/pelvic venous incompetence Premenstrual syndrome/dysphoric disorder Vulvodynia *Hematological* Iron deficiency Mast cell activation disorder	*Neurological* Chiari 1 malformation Migraines Thoracic outlet syndrome Hypersensitivity to light, touch, sound, odors, and medicines *Respiratory* Allergic rhinitis Asthma Chronic sinusitis *Rheumatological* Joint hypermobility syndrome Juvenile fibromyalgia Myofascial pain syndrome Ehlers-Danlos syndrome Temporomandibular joint disease *Sleep disorders* Periodic limb movement disorder Restless leg syndrome *Urinary* Interstitial cystitis

### Cognitive Impairment

Cognitive deficits (sometimes called “brain fog”) are some of the most functionally disabling symptoms of ME/CFS. They are of great concern to children and adolescents because they impact their ability to learn and attend school (see The School System). Cognitive impairments are similar in both adult and young patients with ME/CFS. Slow mental processing speed, impaired working memory, poor learning of new information, difficulty with word retrieval, increased distractibility, decreased concentration and attention span, and inability to multitask may be found (85–87).

The severity of cognitive problems fluctuates together with disease activity both during the day and from day-to-day. Cognitive deficits can be related to abnormal neurological pathophysiology and reduced cerebral blood flow (see Etiology and Pathophysiology).

Any form of mental activity can be followed by diminished cognitive functioning as well as other post-exertional symptoms in a manner similar to the exacerbation following physical exertion. Cognitive symptoms can also be aggravated by a multitude of other factors including: pain (especially headaches), poor sleep, prolonged upright posture, a noisy classroom, eye fatigue from staring at a computer screen for too long, carrying heavy books around school, and social interactions. Young patients might be motivated to keep up with their peers and push themselves mentally, physically, and socially beyond their comfort zone, and suffer cognitive consequences. Cognitive problems can also be exacerbated by the stress of living with a chronic (often undiagnosed) illness (see Psychological Reactions Secondary to ME/CFS: Distinction from Depression and Anxiety Disorders).

### Psychological Reactions Secondary to ME/CFS: Distinction from Depression and Anxiety Disorders

Studies focusing on psychological problems in pediatric ME/CFS patients are limited. In this section, available studies have been supplemented by the insights of our experienced clinicians. It is important to distinguish: (a) normal emotional reactions to ME/CFS from (b) clinically significant psychiatric symptoms such as depression or anxiety secondary to ME/CFS and from (c) a primary psychiatric illness such as Major Depressive Disorder (MDD) or an anxiety disorder without co-existing ME/CFS.

#### Emotional Responses to a Chronic Illness

Emotional responses to the difficulties of ME/CFS are common. These responses are similar to the responses of young patients with other chronic illnesses. Grief and anger can arise over illness-related losses, from negative responses to the illness from family members, friends and school staff, and from the pervasive stigmatization of this particular illness. Apprehension commonly follows the onset of an illness, which can be undiagnosed and about which there is generally ignorance. There can be frustration with the inability to do things that were easily done beforehand. Outbursts of weeping can stem from excessive tiredness and/or feeling overwhelmed. Emotional distress can also occur because of disbelief by others regarding the reality of the illness, or by the prescription of inappropriate remedies by health-care providers.

Some young patients despair of medical care and plead to decline hospital admission, due to previously experiencing the hostile and disbelieving attitudes of some health care providers and/or the exacerbation of their illness by increased hospital-related activity and the noisy environment. These emotional responses to the illness do not ordinarily rise to the level of a psychiatric disorder, but occasionally, psychiatric symptoms are more severe, and a clinically diagnosable, psychiatric disorder can co-exist with ME/CFS.

#### Identifying Clinically Significant Depression and Anxiety Co-Existing with ME/CFS

The most common secondary psychiatric symptoms are depression and anxiety. These symptoms can be present together in the young patient. Clinically significant depression and anxiety can be uncovered by clinical evaluation and the use of standardized questionnaires. Occasionally depression or anxiety predate the onset of the illness, but distinctive, abnormal, pre-illness personality characteristics have not been identified in young ME/CFS patients.

Identifying psychological disorders in younger children can be difficult. They might not manifest depressed mood, as they might not be developmentally able to sense, and verbalize their moods. Rather, they might show behavioral symptoms such as irritability, low frustration tolerance, tantrums, somatic complaints, and/or withdrawal.

Many young patients with ME/CFS are miserable and discouraged (fed up) by being ill, but are not necessarily depressed. There is a higher incidence of clinical depression when the young patient has encountered disbelief in the reality of her/his illness.

In otherwise healthy young people, approximately 2% of children and 4–8% of adolescents have a diagnosable, major depressive disorder (MDD). Less severe episodes of depression can be seen in another 5–10% of children and adolescents. In adolescents, twice as many females as males meet criteria for depression. In one large study of ~400 patients with ME/CFS, 25% had clinical depression using the Beck scale, while the baseline level of depression in the adolescent population was 20% (88).

Raised apprehension levels are a common response to illness. Anxiety secondary to ME/CFS can arise with the illness onset and persist because the illness affects all aspects of the young person’s life. Clinical observation suggests that the incidence of panic attacks might be higher in young patients with ME/CFS than in a normal population of teenagers. Clinical observation has also shown a higher degree of anxiety in patients with co-morbid OI and joint hypermobility. It is postulated that the anxiety might be associated with raised levels of catecholamines and reduced brain blood flow in these conditions.

#### Differentiating ME/CFS from Primary Depressive and Anxiety Disorders without Co-Existing ME/CFS

This can be challenging. Symptoms common to both ME/CFS and psychiatric illness include fatigue, change in activity levels, reluctance to engage in social activities, difficulty sleeping, poor memory and concentration, appetite/weight changes and absence from school.

Some features distinguishing ME/CFS from primary psychiatric illnesses are shown in Table 5. In patients with ME/CFS, long-lasting post-exertional exacerbation of fatigue and other symptoms can follow mild exertion or even normal activity, whereas patients with major depression or anxiety often feel better after increased activity, exercise, or mental effort. OI, hypersensitivities to light, noise and medications and/or low body temperature, and intolerance to heat and cold are typical of ME/CFS, but not typical of psychiatric illnesses. Young women with ME/CFS often experience greater premenstrual mood lability.

**Table 5:** Symptom comparison between depression/anxiety disorders and ME/CFS in children and adolescents.

Symptoms	Depression/anxiety disorders	ME/CFS	Comments
Fatigue, lack of energy, difficulty sleeping, cognitive problems, weight gain, or loss	Yes	Yes	These symptoms occur in both conditions. In ME/CFS, fatigue tends to fluctuate during the day and from day-to-day

Absence from school	Yes	Yes	ME/CFS is the most common medical cause of prolonged absence from school

Depression, feeling sad for no apparent reason	Yes	Sometimes	Young patients with ME/CFS might be sad, discouraged and fed up. Clinical depression is more likely in those who encounter disbelief in the reality of their illness

Anxiety	Yes	Sometimes	In young patients with ME/CFS, anxiety can be associated with having an undiagnosed illness, ignorance about ME/CFS, and/or skepticism about the reality of the illness from family members, physicians, or staff at school. Panic attacks are occasionally seen. There is a higher degree of anxiety in young patients with co-morbid OI and joint hypermobility

Feelings of worthlessness, guilt, low self-esteem	Yes	No	Occasionally, young patients with ME/CFS feel guilty because the illness has caused family disruption. These feelings are secondary to the illness

Anhedonia (lack of interest and/or pleasure in activities previously enjoyed)	Yes	No	Young patients with ME/CFS often wish to engage in previous activities but are physically unable to do so. They might still enjoy previous activities even when the activities exceed their energy limits. Patients with depressive illness do not wish to engage in, or enjoy previous activities, but are physically able to do so

Severe depression with suicidal thinking	Yes	No	Severe depression with suicidal thinking is not present in ME/CFS without co-morbid major depressive disorder

Lack of interest in friendships/relationships	Yes	No	Young patients with ME/CFS often want to socialize, but are physically and cognitively unable to do so. Patients with depressive illness often do not wish to socialize

Post-exertional symptom worsening	No	Yes	This is a hallmark of ME/CFS. Patients with Depression/Anxiety often feel better after exertion.

Orthostatic intolerance (OI)	Occasionally	Sometimes	Much more common in young patients with ME/CFS

Hypersensitivities to light noise, odors, and medications	No	Sometimes	Common in young patients with ME/CFS. Can contribute to feeling anxious and overwhelmed

Difficulty with thermoregulation, low body temperature, and intolerance to heat and cold	No	Sometimes	Common in young patients with ME/CFS

Most teenagers with ME/CFS are highly motivated to recover and return to their previous lives. They generally have a strong desire to be more active, but cannot tolerate the necessary exertion. In contrast, patients with major depression do not have the desire to be more active but could be.

Adolescents with MDD show depressed mood, a sense of worthlessness or guilt, low self-esteem, loss of interest in socializing and in previously enjoyable circumstances (anhedonia), and a lack of interest in the future. Although these are typical symptoms of MDD, some young people with ME/CFS (but without co-morbid depressive illness) might also feel guilty because their illness has disrupted their family, and a parent might have had to give up work to care for them. They might also be reluctant to make plans to socialize, because they know from past experience that they might find themselves at the time of the event to be too ill to participate, have to cancel, and let down their friends. Anhedonia has not been reported in ME/CFS unless MDD was also present. Young people with ME/CFS often take much pleasure in previous activities, even if the activities exceed their energy reserves and cause subsequent symptom exacerbation.

In young people with MDD suicidal thoughts and suicide attempts can occur, more so in adolescents than in younger children. If suicidal thoughts are present a suicide evaluation should be done and referral to a child psychiatrist is often helpful. Suicidal thoughts are only seen in ME/CFS when MDD is also present.

Myalgic encephalomyelitis/chronic fatigue syndrome has a significant impact on a young patient’s parents and siblings. This impact of the illness on family members can have a secondary effect on the patient (see Impact of ME/CFS on the Family). Psychiatric symptoms persisting during the recovery phase of ME/CFS can make it difficult to determine whether the patient’s limitations are due to ME/CFS, or due to associated psychiatric illness.

#### Psychiatric Illnesses That May be Misdiagnosed in Young Patients with ME/CFS

Lack of familiarity with the clinical diagnostic features of ME/CFS and skepticism about its existence has often resulted in a misdiagnosis with one of the following conditions:

- Factitious disorder by proxy (Munchausen syndrome by proxy, fabricated or induced illness) (FDP/MSBP).
- School refusal (school phobia).
- Pervasive refusal syndrome (PRS).
- Somatoform disorder.

#### Factitious Disorder by Proxy (Munchausen’s Syndrome by Proxy, Fabricated or Induced Illness)

In cases of FDP/MSBP, the perpetrator, usually the mother, induces illness in the victim who is often her very young child. [The average age of patients with FDP/MSBP is 4 years old and 50% of patients are <2 years old (89)] Such cases are extremely rare. Most pediatricians only see 1–2 cases in their whole professional careers. FDP/MSBP can be difficult to diagnose but it is a diagnosis that needs to be made as it is a form of child abuse and can result in death of the child.

FDP/MSBP is sometimes falsely diagnosed in young persons with ME/CFS. A report in the press showed that of 143 young persons diagnosed with ME/CFS, but alleged to be victims of FDP/MSBP, (who were assisted by a charity), none were found to be cases of FDP/MSBP (90).

Thus, the treating physician might need to firmly establish the diagnosis of ME/CFS and show how ME/CFS differs from MSBP/FDP. Some distinguishing features are shown in Table 6 (89).

**Table 6:** Comparison between ME/CFS and factitious disorder/Munchausen’s syndrome by proxy/fabricated or induced illness.

	Pediatric ME/CFS	Factitious disorder by proxy^a^
Prevalence	Approximately 100–500 per 100,000	Very rare, approximately 0.4–2 per 100,000

Age and age range	Peak age 10–17 years, range 2–17 years	Average age 4 years; >50% of patients are aged <2 years; extremely rare in adolescents

Symptoms	Consistent pattern of symptoms: reduction in activity, post-exertional worsening of symptoms, fatigue and loss of stamina, unrefreshing sleep, cognitive problems, orthostatic intolerance and headaches; a history of surgeries is uncommon	No consistent symptoms. Most frequently reported are: apnea/cyanosis, anorexia/feeding problems, diarrhea, seizures, asthma, allergy, fevers, behavioral problems, and other sometimes bizarre symptoms, a history of surgeries is common

Clinical signs	Intermittent conspicuous facial pallor, acrocyanosis, joint hypermobility common, blood pressure can fall or heart rate rise on prolonged standing	No consistent clinical signs, many patients have multiple surgical scars

Orthostatic intolerance (OI)	Many patients have POTS or NMH	Not reported

Blood testing	Routine blood tests usually normal	Routine blood tests can show bizarre abnormalities

Neuroimaging	Neuroimaging can reveal defects in brain perfusion and/or metabolism	No reports of neuroimaging

Multiple doctors seen	Common	Common

Hospital admissions	Hospital admissions unusual	Hospital admissions very common

Medical knowledge	Parents can be very knowledgeable about ME/CFS	Parent/perpetrator can be knowledgeable about medical conditions

School absence	Common, it is the most common medical cause of prolonged school absence	Most patients are pre-school age. Common in school-age patients

Separation from parents	Detrimental. Patient does not recover, illness often worsens	Patient improves/recovers when separated from a parent/perpetrator

Mortality	Extremely low	Approximately 10% in patients and 25% in patients’ siblings

*^a^Shaw et al. (89)*.

During history taking, the parents sometimes refuse permission for the clinician to speak to the young person without a parent being present. The young patient and parents might have had previous unhappy or unhelpful experiences with other professionals. Similarly, a young person with ME/CFS may turn to her/his parents when being questioned. This is a natural response in an ill child who has cognitive dysfunction. These behaviors by themselves do not support a diagnosis of FDP/MSBP. Suspicion of FDP/MSBP can result in referral to child protection services (CPS). Young persons with ME/CFS placed in CPS do not fare well. When separated from their parents and sent to live with foster parents or admitted to psychiatric institutions, their ME/CFS symptoms have often worsened.

#### School Refusal (School Phobia)

School refusal has an estimated prevalence of 1–5% and is more common than ME/CFS. It occurs predominately in children less than 12 years of age (91). Children with school refusal fail to attend school because attendance causes emotional distress and anxiety. At the prospect of going to school, the young person exhibits behavioral symptoms, including temper tantrums, crying, angry outbursts, threats to hurt her/himself, and/or a variety of somatic complaints, such as nausea, dizziness, pains, diarrhea, or trembling.

In contrast, young people with ME/CFS usually want to attend school but are prevented from doing so by physical limitations. Other distinguishing features of ME/CFS are post-exertional malaise with exacerbation of other symptoms and OI. Symptoms of school refusal improve once the child is allowed to stay at home and resolve during weekends and school vacations. By contrast, in the young patient with ME/CFS, symptoms persist during weekends and school vacations, but can improve slightly due to decreased activity. During convalescence from severe ME/CFS, the young patient might find it difficult to return to school, having been absent for a variable period of time, having lost contact with many of her/his friends and because of concern those symptoms might worsen. Such hesitancy should not be misdiagnosed as “school refusal.” It should be managed with understanding by the parents, the physician and school personnel.

#### Pervasive Refusal Syndrome

This condition describes a young patient who has despaired of any help from medical care and has lost all hope, as if wanting to die and, therefore, rejects medical care. It is extremely rare. Individual case reports are still being published. The young person refuses food and fluids, and might pull out IV cannulas and nasogastric tubes. It has occurred in young patients with neoplastic disease sickened by too many interventions and it has been described subsequent to sexual abuse. PRS is sometimes wrongly diagnosed in very severe cases of ME/CFS, when the young patient is physically incapable of sitting up or even swallowing. In contrast to PRS, the young person with severe ME/CFS usually wants to get better and co-operates with medical help such as tube feeding. Management of the two conditions consists of the avoidance of stress, medical help with nutrition, assistance with living confined to bed, and an empathetic form of management to which the young person gives consent. A mistaken diagnosis of PRS in a patient with very severe ME/CFS can result in transfer of care to a psychiatrist whose management might include detrimental regimes, such as forced exercise and separation from family.

#### Somatoform Disorders

Somatoform disorders are psychiatric disorders that cause bodily symptoms that cannot be traced back to a physical cause. Prior to diagnosing a somatoform disorder, it is necessary to rule out medical conditions with overlapping symptoms, (such as ME/CFS). Symptoms such as pain occur in both ME/CFS and somatoform disorders, but post-exertional exacerbation of symptoms and orthostatic hypotension are found in ME/CFS, but not in somatoform disorders. Serious deterioration can occur in young patients with ME/CFS who are misdiagnosed as having somatoform disorder and given psychiatric treatment that includes rigidly enforced exercise.

#### Other Psychiatric Conditions

Some additional psychiatric conditions might need to be differentiated from ME/CFS. ME/CFS symptoms such as poor concentration and loss of short-term memory, noticed in the patient’s classroom, can sometimes lead to an erroneous diagnosis of attention deficit disorder without hyperactivity. If the young patient is unable to eat properly due to nausea and gastrointestinal symptoms, ME/CFS must be distinguished from an eating disorder. ME/CFS may also need to be distinguished from substance abuse.

## Management/Treatment

Myalgic encephalomyelitis/chronic fatigue syndrome impacts a young person’s entire life. Coping with debilitating medical symptoms, changed relationships within the family, absence from school, and loss of socializing with peers can all result from the illness. These losses can trigger confusion and crisis. Often the patient might not have been diagnosed and might not have received appropriate help from previous health practitioners, since the young person may not look ill and may have a normal physical examination as well as normal routine laboratory tests.

Given the paucity of peer-reviewed literature on the management of ME/CFS in this age group, this chapter is a compendium of the experience of several clinicians, each of whom has treated children and adolescents with ME/CFS for over 20 years. The chapter provides recommendations primarily for ambulatory patients who are able to attend office visits even if using a wheelchair. Special consideration is given in the section “Severely Affected and Very Severely Affected Young Patients” for young people who are housebound or bedridden.

### Approach to Management

Currently, there is no treatment protocol or intervention which will cure ME/CFS. The role of the physician is, therefore, first, to do no harm, second, to try to improve daily function, expand activity, and ameliorate specific symptoms, and third to support the patient and the family. School personnel often need to be educated about ME/CFS and made aware that ME/CFS is a physical/organic illness, not a psychological disorder. We caution against reliance on internet-based information because much of it is anecdotal, uncorroborated, and may be designed to sell unproven products or services.

Due to variation in symptoms and co-morbid conditions, no single approach works for all patients. Management requires careful attention by the patient, the family and the practitioner to factors that provoke symptoms and a willingness to try several approaches before improvement is achieved.

Most young people can achieve functional improvement. But even in the absence of improvement, the practitioner can help affected young people simply by continuing to evaluate them, providing encouragement, and discussing any new treatments that emerge. A survey of adolescents with ME/CFS found that doctors were considered to be most helpful when they validated the illness, acknowledged its effects, provided ongoing support, and monitored progress (23).

Management is based on: early diagnosis, educating the patient, the family and school personnel about the illness, determining the dominant causes of post-exertional symptoms, treating symptoms with non-pharmacological and pharmacological interventions, providing guidance on activity, diet, maintaining social contacts and educational opportunities, and monitoring progress.

Establishing the diagnosis of ME/CFS and acknowledging that the patient has a recognized illness can produce much relief. In explaining the illness, it is important to reassure the patient that the illness is real despite often normal blood-test findings and the potential for functional improvement should be emphasized. A firm diagnosis can relieve fears of other illnesses such as malignant disease, relieve fears of imminent mortality, validate the presence of symptoms, facilitate the development of a therapeutic partnership with the patient and family, enable the establishment of a management plan, and help organizations such as the young person’s school to provide appropriate education.

Misinformation or absence of information about ME/CFS is common. Educating the patient, the parents, the wider family and school personnel about the illness is important, e.g. providing handouts (Appendices C–E). Sometimes, school personnel and child protection services might need to be persuaded that their misapprehension that the young patient has factitious disorder by proxy (Munchausen’s syndrome by proxy) or pervasive refusal syndrome is wrong and that these mis-diagnoses can seriously harm the young patient.

Clinical management can be improved by the following suggestions:

- Obtain a written list of the patient’s most troublesome symptoms.
- Agree with the patient and family to focus on a limited number of symptoms.
- Recommend that a family member write down or record medical advice.

In reviewing the patient’s most troublesome symptoms, special attention should be paid to those factors which exacerbate symptoms, e.g., upright posture. Then a decision can be made about which symptoms should be addressed first.

Management focuses on non-pharmacological and pharmacological interventions. Medication can be most helpful in the relief of some individual symptoms. Since many pediatric patients with ME/CFS respond to much lower than standard doses of medication (1/2 or 1/4 of the usual dose), the dosage should start low and be increased slowly. In general any change in medication should be made one medication at a time, so that any favorable or unfavorable effects can be attributed to one medication.

Guidance needs to be given on an activity plan that helps the young person to function. Adolescent patients are encouraged to propose a personal, optimal balance of social, physical, and academic activities (and include something pleasurable outside of the home). Very little else is under their control. At each visit, the activity plan can be adjusted, in relation to symptom severity or improvement. Advice on schooling depends on the severity of the illness (see “The School System”).

Successful symptom management requires a regular review of symptoms and symptom severity. Visits every 1–3 months or so, depending on level of severity can be helpful. In patients who are improving, fatigue can be one of the last symptoms to subside. Young people often expand their activity level as they start to improve, preferring to tolerate some symptoms rather than remain on restricted activities so as to experience less fatigue. Thus, measuring improvement by asking solely about fatigue can underestimate progress. Functional improvement can be judged by determining how much activity it takes to provoke post-exertional worsening of symptoms. It can be helpful to use questionnaires/forms which measure activity, cognitive function, and mood to quantify the severity of the illness, and document progress. Brief questionnaires used in some pediatric ME/CFS studies include the Functional Disability Inventory (92), PedsQL (93), and Wood Mental Fatigue Inventory (94). Progress should be measured over months or years, rather than at a single clinic visit.

The young patient should be encouraged to report any changes or additional symptoms as symptoms of ME/CFS wax and wane. The physician might need to determine whether new or changing symptoms represent an altered ME/CFS symptomology, the onset of common co-morbid conditions, or whether new symptoms are suggestive of an alternative diagnosis. Changes to medications and/or their dosages might be required. Reassurance that medical advice from the treating physician will continue to be available can help the patient and family cope with the disease. Telephone or Skype contact can serve the same purpose for those who are more severely affected and unable to travel, or those who are geographically remote.

### The Acute Stage of ME/CFS

In the early, acute, febrile stage of ME/CFS, the diagnosis can be uncertain and other causes of fever need to be considered. Adequate rest and activity management are the mainstays of treatment. Premature resumption of activity or attempts to return to school can result in a relapse or increased severity of symptoms.

### Sleep Problems

Patients with ME/CFS experience unrefreshing sleep. Disturbed sleep patterns include difficulty falling or staying asleep, frequent awakenings, vivid dreams, day/night reversal, and hypersomnia. For many young patients, naps are important to get through the day, but marked daytime sleepiness can result from conditions such as sleep apnea. Sleepiness can be measured using the Epworth sleepiness scale (95).

Hypersomnia (sleeping for up to 20 h a day) can occur in the early stages of the illness and can persist for weeks and occasionally months. Young people who sleep for more than 12 h at a time can develop dehydration. During wakeful periods, the young patient’s parents/caregivers need to ensure adequate hydration and nourishment. When oral hydration and nourishment are inadequate, tube feeding is necessary. If hypersomnia persists for months, the young person should be evaluated by a sleep specialist, preferably one who is familiar with ME/CFS.

Conventional sleep hygiene measures for insomnia in otherwise healthy people can be ineffective in young people with ME/CFS, but the following measures can be helpful:

- Balance daytime activity with rest (pacing of activity), to avoid post-exertional symptom exacerbation, which can interfere with sleep (see Fatigue, Post-Exertional Symptoms, Exercise Intolerance).
- Eliminate caffeine-containing beverages in the late afternoon and evening.
- Avoid television, computers, and electronic devices before and after bedtime, (light from electronic devices can aggravate insomnia and fatigue).
- A carbohydrate snack at bedtime might be helpful (96).

If insomnia or sleep reversal is profound, sleep medications, can be started at a low-dose. The risk of adverse effects needs to be balanced with the gains from a better night’s sleep and sleeping the same hours as other family members. The medications chosen should be safe for long-term use and should be taken early enough to be effective at bed time. Non-pharmacological remedies can include herbal teas, such as Chamomile. Commonly used medications are listed in Table 7. For more severe problems, Zolpidem and trazodone can be used with caution. Controlling pain (see Pain) will often help sleep.

**Table 7:** Medications for sleep.

Medication	Usual adolescent dose	Comments
Melatonin	3 mg (range 1–12 mg)	Use dose that is effective given half to 1 h before bedtime
Diphenhydramine	25 mg at bedtime	Can cause excessive sedation or dry mouth
Tricyclics (TCAs) Amitriptyline Dothiepin	10–25 mg at bedtime 25 mg at bedtime	Can be helpful for pain and headaches. Can cause weight gain, dry mouth, lightheadedness, and excessive sedation
Clonazepam	0.5–1 mg at bedtime	Helpful if anxiety is affecting sleep. Can help restless leg syndrome. Using low doses, taken only at night, physiological dependence is unlikely

### Pain

Pain in ME/CFS can be widespread or localized. Sometimes pain is so severe that the patient is unable to tolerate even a gentle touch. Common types of pain include: headache, abdominal pain, myalgia, joint pain, sore throat, lymph nodes, eye pain and occasionally pelvic pain or dysuria (97, 98). It is important to treat localized pain such as headache, because pain can amplify other symptoms.

Chronic, daily headaches, which can fluctuate in severity from week-to-week, are common. If they are episodic, a diagnosis of migraine should be considered. Possible triggers include inadequate sleep, stress, skipped meals, specific foods and supplements (including, but not limited to chocolates, nuts, and aspartame). Initial intervention focuses on avoiding the common triggers. Migraine-prevention drugs are worth a trial for both episodic and non-episodic chronic, daily headaches. Commonly used medications are shown in Table 8. Beta blockers can help headaches that are associated with OI (see Orthostatic Intolerance).

**Table 8:** Medications for headaches.

Medication	Usual adolescent dose	Comments
**Supplements**		
Riboflavin	200 mg bid or 400 mg daily	Available as a single supplement or in combination with others (e.g., MigreLief)
Co-enzyme Q10	300 mg daily in 2–3 divided doses	
Magnesium	250 mg daily	
**Antihistamines**		
Pizotifen	1–1.5 mg nightly	Helpful for co-morbid insomnia and for poor appetite. Can cause weight gain. Pizotifen is not available in all countries
Cyproheptadine	2–8 mg nightly	
**Tricyclics**		
Amitriptyline	10–25 mg nightly (Up to 50 mg may be tolerated)	Useful for co-morbid fibromyalgia and abdominal hyperalgesia. Can cause lightheadedness and fatigue even at low doses. Can take 4 weeks to become effective.

Myalgia and pain from co-morbid fibromyalgia can be found in from 10 to 30% of pediatric patients with ME/CFS and is less common than in adults (98). Abdominal pain and Nausea are common (98, 99). Abdominal hyperalgesia may also occur. Gastrointestinal motility disorders can be present, especially in patients with OI.

Helpful, non-pharmacological remediation for pain includes: pacing of activity to avoid flare-ups (see Fatigue, Post-Exertional Symptoms, Exercise Intolerance), hot or cold packs for treatment of tender points, warm baths, muscle liniments, physical therapy, transcutaneous electrical nerve stimulation, acupuncture, massage, yoga or Tai Chi, biofeedback, and mindfulness-based stress reduction techniques. These interventions might not be effective in some patients and might be poorly tolerated in others.

For persistent pain, medications might be needed. Over-the-counter medications such as aspirin, acetaminophen, paracetamol and NSAIDS are rarely effective for ME/CFS pain, but NSAIDS can ameliorate dysmenorrhea. Topical treatment for pain includes lidocaine patches. Some commonly used prescription pain medications and their dosages for adolescents are listed in Table 9. We recommend beginning at a low-dose and increasing very cautiously. If pain is generalized, the higher dosages might be needed. Multiple trials of medication might be necessary to achieve adequate relief.

**Table 9:** Medications for generalized pain.

Medication	Usual adolescent dose	Comments
Tricyclics		See Table 7
**Selective serotonin reuptake inhibitors or SNRIs**		
Sertraline HCl Escitalopram Venlafaxine HCl Duloxetine HCl Bupropion HCl	25–100 mg daily 5–20 mg daily 37.5–225 mg daily 20–60 mg daily 150–300 mg daily	Consider in patients with co-morbid anxiety or depression. These medications should not be stopped suddenly. Patients should be weaned very gradually off them, especially duloxetine. Duloxetine can be helpful for fibromyalgia
**Anticonvulsants**		
Topiramate (Topamax)	25–100 mg bid	Helpful for widespread pain, continuous headaches, and fibromyalgia. Can cause weight loss
Gabapentin	300–1,200 mg tid	Adverse effects of topiramate include suicidal feelings and cognitive problems with doses >50 mg daily
Pregabalin (Lyrica)	25–50 mg at night increasing to 25–100 mg tid	Gabapentin can cause somnolence and weight gain
Valproic acid	250–500 mg bid	

Young people with severe pain sometimes need strong analgesics. Opiates are occasionally necessary. Their use requires full documentation. For young people with complex pain syndromes, referral to a pain clinic (preferably one that is familiar with ME/CFS) might be helpful. We have received anecdotal information from some young patients that their chronic pain was improved by the use of cannabis or synthetic cannabinoids. There have been no clinical trials of this medication.

### Fatigue, Post-Exertional Symptoms, Exercise Intolerance

Myalgic encephalomyelitis/chronic fatigue syndrome is characterized by the body’s inability to produce adequate energy for the normal range of human activity. Patients with ME/CFS experience persisting, overwhelming, physical and cognitive exhaustion that is not relieved by rest or sleep. There is a loss of physical and mental stamina. Sometimes there is a feeling of being tired and wired (overstimulated). Fatigue in ME/CFS is pathological. It is qualitatively and quantitatively different from the normal fatigue experienced by healthy people following exertion which is relieved by a good night’s sleep.

Following even minor physical, cognitive or orthostatic activity, the fatigue and other ME/CFS symptoms are worsened. This worsening of symptoms can occur any time from immediately following activity, to up to 48 hours following activity and it can last for days or weeks or months. This phenomenon is the cardinal feature of ME/CFS.

Although the overall mechanism for triggering post-exertional symptoms is currently unknown, fatigue, post-exertional symptoms and exercise intolerance in ME/CFS might be related to defective energy metabolism. Aerobic metabolism has been shown to be impaired in adult patients with ME/CFS (100). This impairment leads to an increased reliance on anaerobic metabolism which is much less efficient at producing energy.

#### Activity and Exercise

Activities of daily living, education and social contact related activities can comprise a tolerable amount of energy expenditure for some patients, but these activities can comprise an excessive amount of activity for others. Adding an exercise program to the schedule of a young person who can barely manage to cope with limited educational activities can be counterproductive. Exercise has been promoted as therapeutic in patients with ME/CFS, but many patients drop out of exercise studies because exercise clearly made them worse (101). Some individuals with ME/CFS mistakenly over-exercise in an attempt to reduce fatigue and then incur post-exertional relapse. No studies have shown that exercise can produce a cure for ME/CFS.

Occasionally an excess of caution in young patients and/or their care givers can result in too much rest. Prolonged periods of complete bed rest should be avoided except in the most severely affected patients. Studies in adults have shown that 2 weeks of complete bed rest can be followed by a substantial reduction in plasma volume, and by OI (102). Striking the right balance between rest and activity while avoiding post-exertional symptoms requires trial and error.

However, for some patients with less severe ME/CFS, or during a remission, a suitable exercise program can improve function and provide some enjoyment. Any exercise program should not take priority over activities of daily living, education and socialization. Special care also needs to be taken to ensure that exercise is not advanced too rapidly or too soon, as by definition, excessive exercise can exacerbate ME/CFS symptoms.

#### Pacing of Activities and the Energy Envelope

Young people with ME/CFS often do better when they (and their parents) learn to adapt their lifestyles to live within their capabilities, and pace or spread out their activities so that they can avoid a boom and bust cycle of over-activity on a given day followed by a “crash” the following day. Remaining as active as possible while avoiding post-exertional flare-ups delineates an optimal zone of activity termed the “energy envelope.” It has been shown in adults that fatigue severity declines when patients stay within their energy envelope (103).

Activities can be planned by the young patient over a weekly period. She/he should be encouraged to balance intellectual, social and physical activities, and to make a commitment to undertake segments of each component regularly. This allows the patient and family members to regain some control over their lives. It must be remembered that activity levels fluctuate from day-to-day and patients sometimes experience set-backs in their available energy reserves. Family members need to recognize that set-backs can occur and any activity plan needs to be flexible.

#### Recommendations for Improving Activity Levels

Guidelines for exercise in healthy, but sedentary young people are inappropriate for patients with ME/CFS, because strict adherence to these guidelines can cause post-exertional relapse. For some young patients, it might be necessary to first treat their OI and improve their ability to remain upright before any exercise can be adequately tolerated. Consultation with a physical therapist or rehabilitation specialist knowledgeable about ME/CFS is often helpful.

##### For the Most Impaired Young People

Advice for those who are homebound or confined to bed can be found in Section “Severely Affected and Very Severely Affected Young Patients.”

##### For the Moderately Impaired

Exercise while lying down should be advised when exercise while sitting or standing is poorly tolerated. Manual forms of physical therapy to improve mobility can be a bridge to tolerating exercise without prolonged exacerbations, especially for those with impaired range of motion on examination (104). Exercise should begin with as little as 1–2 min of gentle stretching followed by rest. When recovery has occurred another 1–2 min can be attempted. When stretching exercises do not trigger post-exertional symptoms, intervals of recumbent exercise can be added such as leg lifts while supine, or the use of a recumbent stationary bicycle. The individual can be encouraged to increase the duration of activity very gradually, provided that the prior period of activity has not aggravated symptoms, until a reasonable exercise volume has been achieved. Rest between activities is needed, and young people are advised to avoid the “push-crash” cycle of excessive activity on a good day followed by prolonged post-exertional collapse. Exercise in a swimming pool is sometimes better tolerated, provided the water is not too warm, as there is an external counter-pressure from the water that can improve circulatory function.

##### Mildly Impaired Patients

Leisurely walking with an initial duration of 5–15 min followed by a rest is suggested. The duration or pace of the walking can be increased gradually provided post-exertional symptoms do not occur.

##### Higher Functioning Patients

An exercise program might involve joining some organized sporting activities at school, but with modified participation. The effect of exercise on ME/CFS should be discussed with the student, her/his parents and the school personnel. Everyone should be reminded that when the patient feels that she/he has done enough, she/he *must* stop and rest and never force her/himself to achieve more. Fluctuations in illness severity are also common and patients might find that they need to reduce their activities for a period of time.

#### Medications for Fatigue

Trials of medications for fatigue have not been conducted in children and adolescents with ME/CFS. A limited number of controlled trials have been conducted in adults. Dexamphetamine, 5–10 mg twice daily for 4 weeks was tested in 20 adults with ME/CFS. The dexamphetamine group had a significant improvement in Fatigue Severity Scale scores at the end of the trial (105). Methylphenidate 10 mg twice daily for 4 weeks was tested in 60 adults with ME/CFS. Compared to placebo, it reduced the severity of fatigue and concentration problems (106). Lisdexamphetamine was helpful for executive function, fatigue and pain in adults with CFS in a small randomized trial (107). Modafinil has been shown to be effective for fatigue in adults with multiple sclerosis. When tested in 14 adults with ME/CFS the treatment effect was uncertain, but these data need to be viewed with caution due to the small number of patients tested (108).

Low vitamin B12 levels can be associated with fatigue, cognitive problems, balance difficulties, and hypotension. This has led some clinicians to administer vitamin B12 injections to adults with ME/CFS in an attempt to improve symptoms and function. The typical dose used is 1,000 μg IM weekly for 4 weeks to assess the response and estimate the duration of effect (typically 3–7 days). A patient survey of treatment rated results as good by some patients (109). There have however been no randomized placebo controlled trials of vitamin B12 for ME/CFS in young people.

Many patients self-medicate with caffeine in coffee and popular energy drinks. While this can be useful, patients should be cautioned against consuming excessive amounts of caffeine as this can cause tachycardia and agitation.

Medications for fatigue might need to be reserved for potentially exhausting occasions such as school exams. If effective, the young person should try to avoid exceeding their individual energy envelope.

### Cognitive Impairment

Cognitive difficulties are an integral part of ME/CFS and they have a significant impact on schooling. Advice on educational accommodations is given in Section “The School System.” The young patient needs to recognize issues that aggravate cognitive difficulties and when possible, avoid them. Aggravating factors include mental and physical activity; distraction by noise or bright lights; and prolonged standing.

Cognitive problems can be improved by:

- Pacing of activities: the young person should be mentally active for short periods of time, followed by adequate periods of rest; she/he should learn to recognize when she/he is tired.
- Performing mental work lying down can sometimes be better than sitting up.
- The effective treatment of insomnia, pain, depression, and/or anxiety.
- Snacks and frequent drinks.
- Self-medicating with caffeine-containing drinks.
- The use of prescribed stimulants such as low-dose methylphenidate can benefit some patients; the patient should be warned that there is a risk that a sense of well-being can lead to over-activity.
- Learning to cope with the stress of having a chronic illness (see Support and Coping Skills).

### Immune System Support

A limited number of trials of intravenous immunoglobulin (IVIG) have been undertaken in adults, with mixed results. In adolescent patients a double blind placebo controlled randomized trial of IVIG 1 g/kg monthly (max dose 60 g) for 3 months was found to have moderate effectiveness for overall function (a combination of school attendance, school work, social activities, and physical activities). Patients also reported general improvements in all ME/CFS symptoms, many reporting feeling completely well by the 6-month follow-up. The placebo group also improved but more gradually, matching the functioning of the intervention group after 5–7 years of follow-up. The trial showed that the benefit from IVIG was in reducing the duration of illness (42). However, IVIG is relatively expensive, is not always available due to health insurance restrictions and other limitations and it can be associated with serious adverse effects, e.g., aseptic meningitis after infusion, and anaphylaxis in those with IgA deficiency (110).

### Depression and Anxiety

Mild or moderate secondary psychiatric problems often can be managed by the primary care physician, provided the physician feels comfortable with informal counseling. Giving support and encouragement to the patient can be adequate treatment. Various forms of support and psychotherapy helpful for learning to cope with the illness are given in Section “Support and Coping Skills.” When referral to a child psychiatrist or psychologist is needed, it is likely to be more successful if she/he is familiar with ME/CFS.

The treatment of other symptoms of ME/CFS such as pain or insomnia can also help to relieve emotional distress. If antidepressant medications are indicated, it should be noted that young patients with ME/CFS often respond to a smaller than expected dosages of these medications and careful follow-up is important.

### Support and Coping Skills

The young person with ME/CFS needs to learn to adapt to the reality of the illness, and integrate it into a meaningful life despite sometimes severe physical limitations. Above all, the young patient needs to develop a sense of achievement in her/his life, however, small.

All aspects of the young person’s life might need to be addressed. She/he might need to deal not only with physical and cognitive limitations, but also with misunderstanding of the illness, fear, grief, anger, guilt and isolation. Sometimes having ME/CFS can result in abnormal illness behavior, such as denial of the reality of the illness.

A patient’s needs early in the illness might differ from her/his needs in later years, as health improvement is being achieved. Young patients should be encouraged to verbalize their fears and needs. Only the young person her/himself knows how she/he really feels. For instance, many young people fear getting behind their peers academically, never being able to catch up, and consequently losing friends. There should be opportunity to talk things through with a trusted professional who understands the illness. Although the parents can be present, the discussion should be primarily with the patient, so that she/he is also involved in decision making and feels part of the team approach. Teenagers usually need an opportunity for discussion without a parent present.

Our experience suggests that the following elements of basic supportive therapy can be helpful:

- Exploring what the patient and her/his parents already know about the illness.
- Teaching the patient and her/his family about ME/CFS and correcting any misinformed notions about cause, course of the illness and forms of management.
- Making it clear that ME/CFS is a medical/biological illness, but that often there can be understandable, secondary, emotional reactions.
- Clarifying and discussing the patient’s and family’s particular anxieties and mood changes.
- Discussing strategies such as pacing of activities (see Fatigue, Post-Exertional Symptoms, Exercise Intolerance) that enable the patient to live within the limitations of the illness.
- Ensuring the medical management of symptoms.
- Making clear that you (as the treating physician and/or therapist) will be available as new questions arise and setting up follow-up appointments.

These steps can be carried out by the primary care physician if she/he is comfortable doing so. If not, or if the patient’s situation is complex or severe, a social worker, psychologist, or child psychiatrist, preferably one who is knowledgeable about ME/CFS, can be consulted.

#### Coping Skills Training

Young patients with ME/CFS benefit from practical suggestions for coping with chronic illness. For example, it can be helpful to provide strategies for demystifying the illness and explaining it to other people: ME/CFS is like having mononucleosis (glandular fever) and then not recovering from it.

We recommend:

- A private space at home, so that the young patient’s rest will be undisturbed and she/he is able to work without being distracted.
- Developing a daily routine, especially for patients who are housebound.
- Attempting to achieve a normal day/night cycle, to help the patient fit in with the family’s daily schedule and make school possible.
- Encouraging participation in education, however minimal, but without undue pressure (see The School System).
- Providing time for meeting with friends and for family treats.
- Incorporating rewards for achievements.
- Joining a local patient support group (if available, and if it has competent leadership).
- The use (when appropriate) of stress reduction techniques such as music, visualization, self-hypnosis and/or mindfulness-based cognitive therapy (111).

#### Cognitive Behavioral Therapy (CBT)

The benefit of CBT as a psychotherapeutic intervention for ME/CFS is currently ambiguous. CBT has been shown to be helpful for some pediatric ME/CFS patients (112–115). However, in adults, the effect size of CBT is rather modest and the effects are not always sustained (116). CBT can produce adverse effects (101). The assertion that CBT can “reverse” or cure the illness is not supported by follow-up studies (117) and immunological changes found in the illness have been shown not to be reversed by CBT (118).

The hypothesis underlying the rationale for the use of CBT in ME/CFS patients proposes that the patient’s poor health is perpetuated by avoidance of activity and by maladaptive fears, such as anxiety about symptoms that emerge after activity (119). CBT aims to improve coping with the illness by changing these “maladaptive cognitive responses” and encouraging graded exercise as well as incremental increases in other activities. The notion that the illness is perpetuated solely by dysfunctional attitudes and beliefs is speculative, lacks empirical support and is not consistent with our current understanding of the pathophysiology of ME/CFS (see Etiology and Pathophysiology). This hypothesis has led to patients being blamed for failure to recover, because, in the view of the therapist, they have not changed their so called “maladaptive cognitive responses.” Furthermore, CBT that includes rigidly enforced graded exercise frequently leads to severe relapse in patients with ME/CFS.

Nevertheless, some of the elements of CBT are part of a common sense management of many medical problems, provided they are introduced in a pragmatic and flexible manner to help individuals cope with chronic illness (113–115, 120, 121). These elements include recommendations already given in this section. It is most important for a patient with ME/CFS that any attempt to increase her/his activity level or to exercise be flexible, rather than rigid, permitting the patient to avoid exceeding her/his energy limit. The patient should also be advised that CBT does not cure the illness, but it can help in learning to live within its limitations. CBT is not always available and it can be costly. It is likely to be more successful if the therapist is knowledgeable about ME/CFS.

### Dietary Management

This section discusses nutrition in patients who are able to take adequate nourishment by mouth. Patients unable to maintain adequate nourishment by mouth are discussed in the section on the severely affected (see Severely Affected and Very Severely Affected Young Patients).

Many young people with ME/CFS experience a variety of gastrointestinal symptoms that can interfere with nutrition. Common complaints are: lack of appetite, especially in the morning, nausea, abdominal pain that might or might not be associated with eating, heartburn, feeling “full” even after a small meal, abdominal bloating, and discomfort that can be associated with particular foods, e.g., dairy or wheat. Co-morbid gastrointestinal conditions are present in some young patients. These are discussed in the section “Gastrointestinal Issues.”

Reduced food intake can stem from food fads. The young person might be a fussy eater, might claim to be “allergic” to almost all foods, or might be fearful that certain foods make the illness worse. Some young people with ME/CFS have a very disordered body clock—up all night, asleep all day—making mealtimes very awkward. As a result of these problems, some young people with ME/CFS develop weight loss. Alternatively, others become obese due to lack of exercise or increased “snacking” for an energy boost, from boredom, or occasionally due to increased appetite from medications. If there is no logical explanation, weight changes should be investigated.

Parental concern and pressure to eat more can contribute to stress in the family. The goal is for the young person to get a balanced nutritional diet, including food they enjoy, with plenty of variety. Educating the patient about how the body needs fuel to function and provide energy can be helpful.

We recommend

- Regular, small meals, little and often, and between meal snacks.
- Plentiful salt for those with co-morbid OI.
- Avoid too much fluid with meals if experiencing bloating.
- A carbohydrate snack at bedtime or if wakeful during the night can encourage sleep.

Extra foods that can be incorporated in the diet in some circumstances include: yogurt, if the illness began with or is complicated by gastrointestinal infection, ginger drinks for nausea, peppermint for indigestion, or garlic to help overcome/prevent infection. Evidence for these being helpful is anecdotal. Snacks should be readily available and nutritious, but include some treats. In school, permission might be needed to have snacks and drinks readily available, particularly during long exams.

Fluid intake should be liberal:

- Drinks should be readily available, water is ideal, do not “over-drink.”
- Electrolyte drinks can be helpful, avoid those containing fermentable oligo-, di-, mono-saccharides and polyols (FODMAPs) (see “Supplements”).
- Avoid alcohol and too much caffeine (coffee, cola, energy drinks).

#### Supplements

If the diet is good, supplements should not be needed, although sometimes they can be beneficial. Occasionally, an excess of supplements can cause problems e.g., too much vitamin C can lead to diarrhea. The following supplements may sometimes be useful:

- Vitamin D if patient lacks sunlight, due to light sensitivity or if seldom outside. Low vitamin D levels can be associated with headaches and pain and these symptoms improve with raising vitamin D blood levels.
- Magnesium at bedtime can help with pain and cramps (take with apple juice or apple to aid absorption), it also helps relieve constipation.
- Vitamin B12 injections can help with low energy/brain fog, or vitamin B12 can be taken as sub-lingual tablets.
- Probiotics can help irritable bowel symptoms, or if taking antibiotics.
- Iron tablets for iron deficiency with/without anemia (the cause of anemia should be determined), take with citrus juice to aid absorption.

Recent attention has focused on dietary FODMAPs. These are short chain carbohydrates (oligosaccharides, disaccharides, monosaccharides and related alcohols) that are commonly found in the modern western diet and are poorly absorbed in the small intestine. They include short chain polymers of fructose (fructans), galactose (galactans), disaccharides (lactose), monosaccharides (fructose), and sugar alcohols (polyols) such as sorbitol, mannitol, xylitol and maltitol. There is evidence that restriction of FODMAPs can have a beneficial effect for those with irritable bowel syndrome and other functional gastrointestinal disorders. It might, therefore, be useful to restrict the dietary intake of these types of carbohydrates for ME/CFS patients with co-morbid gastrointestinal symptoms.

### Alternative and Complementary Medicine

Many alternative and complementary therapies have been promoted for ME/CFS. It is difficult to determine whether these therapies actually work, because the symptoms of ME/CFS vary so much from day-to-day. Anecdotal evidence suggests that acupuncture, massage, pilates, and yoga can help pain in some adults, but no published studies have assessed their benefit in young people.

The clinician should identify the use of herbal/natural remedies or supplements. The contents of complementary medicines are not regulated for dose or composition. Caution must be exercised regarding side effects because if used with prescribed medications, there can be interactions.

Unfortunately, in the hope of a cure, parents of young patients with ME/CFS often ask the young patient to try costly, non-established and speculative treatments but find little or no clinical improvement. Feedback from young people with ME/CFS indicated that 80% had tried up to 30 different alternative therapies. Only massage for pain relief and “good dietary advice” achieved “some benefit” in up to 30% of patients. A common comment from the patients was that they “were glad when their parents stopped shopping around for a cure.” (23). A review of alternative medicine studies in adults with ME/CFS revealed generally poor methodologies and limited evidence of any benefit (122, 123).

## Co-morbid Conditions

Co-morbid conditions are seen frequently in young patients with ME/CFS and are major contributors to illness severity. Their successful management can result in substantial lessening of the burden of illness. A list of co-morbid conditions is given in Table 4. This section will discuss those co-morbid conditions that can have a significant impact on the illness, including orthostatic intolerance, joint laxity, gynecological problems, gastrointestinal problems, allergies, intolerances and neuroanatomic abnormalities.

### Orthostatic Intolerance (OI)

The term OI refers to a group of conditions in which symptoms worsen with quiet upright posture and are improved but not always abolished by lying down. Typical symptoms are those of cerebral hypo-perfusion or sympathetic activation. Studies report higher rates of OI in pediatric patients with ME/CFS than in healthy children and higher rates than in adult patients with ME/CFS. An estimated 60–95% of young patients with ME/CFS have orthostatic symptoms (49). OI is more common in girls after puberty (sex ratio 3:1). OI can follow an infectious illness or an immunization.

Because there are more patients needing help with the management of OI than specialists trained to meet their needs, we have included the salient points of diagnosis and management of OI in this section. There is more detailed information on the pathophysiology and testing for OI in Appendix G.

Symptoms of OI in young patients can precede the onset of ME/CFS or they can arise a period of time after the onset of ME/CFS. Some young patients with symptoms of OI do not develop symptoms of ME/CFS, while in others, the concurrent diagnosis of ME/CFS has been missed, because symptoms such as post-exertional worsening have not been asked about.

Orthostatic symptoms include any of the following: increased fatigue, lightheadedness, white-outs or black-outs of the visual field, visual dimming, mental fog, headaches, nasuea, pain, or shortness of breath. Upright posture consistently aggravates ME/CFS symptoms and patients report worsening fatigue and other symptoms while standing in line, in hot environments like a shower, or in the summer heat. Many patients adopt postural counter-maneuvers—such as sitting with knees to chest, doing homework in a reclined position, crossing the legs when standing, fidgeting in line—but are not aware of why they have done so. Some adolescents might not report lightheadedness, so asking about symptoms that emerge during prolonged upright posture can be revealing.

Characteristic physical appearances include facial pallor and a reddish-purple discoloration of the dependent limbs (acrocyanosis) when sitting or standing for more than a few minutes. Symptoms of OI can occur without prominent changes in heart rate and blood pressure, but are often associated with objective circulatory disorders. Postural tachycardia syndrome (POTS) is the most common, neurally mediated hypotension (NMH) is less common, and orthostatic hypotension (OH) is uncommon in pediatric patients. The detection of POTS, NMH, and OH require a prolonged period of orthostatic stress and they can be missed with brief duration 1–2 min orthostatic vital sign measurements. We recommend a 10 min standing test in all young people with ME/CFS to ascertain whether orthostatic symptoms occur and determine whether POTS and/or OH are present (Table 10). Patients with NMH are generally symptomatic soon after standing, but longer duration tilt table testing is required to elicit the hypotension. Tilt table testing requires referral to a specialist center and is costly.

**Table 10:** Orthostatic testing.

In-office standing tests
Five minutes supine, then at least 10 min of quiet standing, leaning against a wall, with instructions to the young person to remain still, not fidget or shift her/his weight. Changes in HR and BP should be recorded each minute, supine and upright, along with intensity of orthostatic symptoms and fatigue on a 0–10 scale. The patient must be carefully observed due to the risk of syncope. The development of pallor, warmth, and/or nausea can be prodromal signs of hypotension or syncope. This test will identify POTS and orthostatic hypotension (OH), but is usually not sufficiently prolonged for neurally mediated hypotension (NMH)

**Head-up tilt table tests**

HR and BP are measured supine and during 70° head-up tilt. POTS or OH can be identified by 10 min tests. Prolonged testing of 40–45 min might be required to identify NMH

*For further details on orthostatic testing and standing test data sheet see Appendix G*.

*POTS*: In adolescents, this diagnosis requires the reproduction of orthostatic symptoms together with a 40 bpm change in HR, from supine to 10 min upright, or a HR of ≥120 (124). Some individuals who have POTS early in upright posture go on to develop NMH if the orthostatic challenge is prolonged beyond 10 min.

*Neurally mediated hypotension* requires the production of orthostatic symptoms with a 25 mm Hg drop in systolic BP, usually without an increase in HR, and can be associated with junctional rhythm (recognized by a loss of P waves on the EKG) at the time of pre-syncope or syncope. The terms vasovagal syncope, neurocardiogenic syncope and NMH are synonymous. Syncope need not be present to make the diagnosis of NMH, as many affected individuals with lightheadedness and other symptoms sit or lie down before fainting.

*Orthostatic hypotension* is defined by a BP reduction of at least 20 mm Hg systolic or 10 mm Hg diastolic within the first 3 min of upright posture (124). This problem is rarely seen in pediatric patients except at times of hypovolemia, such as febrile illness, acute dehydration, hemorrhage, adrenal insufficiency, or excessive histamine release.

#### Management/Treatment

The lack of treatment studies in young people with OI and the lack of specialists with experience in OI contribute to difficulties in managing this condition. Some young patients have characteristic symptoms of OI, but at the time of testing have a standing HR rise or BP fall which is insufficient to diagnose POTS, NMH, or OH. They might still benefit from treatment.

*The first step* in management is non-pharmacological and emphasizes four main points: (a) avoid conditions that increase pooling of blood, (b) improve venous return to the heart, (c) avoid depletion of salt and water and other causes of low blood volume, and (d) avoid increasing catecholamines beyond their baseline levels (which can be elevated).

##### Avoid Pooling of Blood

This involves avoiding prolonged standing or sitting, such as by moving around during longer classroom lectures, standing and stretching periodically to break up study sessions, and shopping at off hours. Patients should avoid saunas, hot-tubs and sunbathing, and take short, cool baths, and showers. Large meals and high carbohydrate intake can interfere with orthostatic tolerance by contributing to a shift of blood volume to the splanchnic circulation. Small, frequent meals are often better tolerated.

##### Improve Venous Return to the Heart

Adolescents can utilize the muscle pump of the lower limbs by e.g., crossing their legs and shifting from one leg to the other while standing, sitting with their knees higher than their hips, or with their knees to their chests, or by performing leg muscle contraction exercises before standing. Sitting on a high stool with the legs dangling freely should be avoided, as there is no resistance to blood pooling in the legs. Some adolescents find they can sit longer without symptoms if they put their feet on a low foot rest, or sit with one leg folded under the buttocks.

Compression garments such as support hose with 20–30 mm Hg compression can be helpful (waist-high garments are more effective than thigh-high, which are more effective than knee-high). Some adolescents derive benefit from wearing body shaper garments or abdominal binders.

A time-honored recommendation to improve blood volume is to elevate the head of the bed slightly by 10–15^o^. While this is not comfortable for everyone, it can help the body retain fluid at night (125, 126).

##### Avoid Depletion of Salt and Water

Patients need to drink 2–3 liters of fluid daily and take in adequate sodium. We recommend drinking fluids every 2 h during the day. There is no specific amount of sodium that works for each individual. Food should be salted according to taste and supplemental buffered salt tablets should be considered if needed. Oral rehydration fluids can also be beneficial. Healthy higher sodium food options include dill pickles, olives, tomato juice, soups, salsa, salted nuts, and soy sauce.

##### Avoid Increasing Catecholamines

Epinephrine (Epi) and norepinephrine (NE) levels are increased in those with OI and worsen with upright posture. Physiological stressors, including pain and emotional distress, can elevate catecholamine levels even higher. Stress avoidance can help with symptom management.

Examine whether medications are helping or making symptoms worse. For example, in those with asthma, beta-adrenergic agonists like albuterol and salbutamol mimic the effects of Epi, and can contribute to tremulousness and lightheadedness in patients with OI. While beta-agonists are not completely contraindicated, we try to use inhaled glucocorticoids, sodium chromoglycate, or montelukast for asthma control. Medications that promote vasodilation, such as niacin, phenothiazine anti-emetics and narcotic analgesics are better avoided or minimized. Although low doses of tricyclic antidepressants used for headache, pain, and insomnia might be tolerated, higher doses can aggravate hypotension.

Caffeine intake (including soft drinks or coffee) can help symptoms by acting as a vasoconstrictor, but some patients experience adverse effects. Alcohol consumption usually aggravates OI symptoms.

*The second step* in management is to treat other ME/CFS symptoms and co-morbid conditions. Treating symptoms, especially pain and sleep problems, can improve OI symptoms.

*The third step* in management is pharmacological intervention, aiming for monotherapy, but often rational poly-therapy produces better symptom control. All medications should be started at low doses and increased very slowly. Some medications are listed in Table 11. Some physicians recommend a low-dose beta blocker or midodrine as the first-line agents. Alternatively an initial medication can be selected from Table 11 depending on the specifics of the patient’s condition and existing co-morbidities. For example: beta blockers might be chosen for those with elevated supine HRs, fludrocortisone might be chosen if there is a low resting BP or an increased salt appetite. Midodrine is efficacious in treating syncope, but 4 hourly dosing makes it less convenient to take when in school. Stimulants can be helpful in those with fatigue and prominent cognitive symptoms.

**Table 11:** Medications for treating orthostatic intolerance (OI) in adolescents.

Medication	Usual dose for OI	Comments
**Vasoconstrictors**		
Midodrine	Start at 2.5 mg q 4 h while awake. Increase every 3–7 days by 2.5 mg to optimal dose or max. of 10 mg q 4 h while awake	First-line therapy for those with low blood pressure (BP) (SBP < 100 or low normal BP). Monitor BP as supine hypertension can occur. Avoid prior to bedtime

Methylphenidate	Immediate-release form: 5–10 mg bid, increasing gradually to 15–40 mg/day	First-line therapy for those with prominent cognitive dysfunction, personal or family history of ADHD, joint hypermobility. The optimum dosage of dextroamphetamine is usually half the optimum dose of methylphenidate

Dextroamphetamine	Sustained-release form: 5–10 mg q AM. Increase by 5–10 mg q AM weekly until optimal dose or 15–40 mg daily	

**Volume expanders**		
Sodium chloride	Oral: 1 g tablets with meals IV: 1–2 L over 1–2 h	IV saline is seldom practical over the longer term, but can help restore baseline function after illnesses or as emergency “rescue” therapy

Fludrocortisone	0.05 mg daily for 1 week, then 0.1 mg daily. Increase gradually to maximum of 0.2 mg daily	First-line therapy in those with baseline hypotension or increased salt appetite. Add KCl 10 m Eq for every 0.1 mg fludrocortisone to prevent hypokalemia

Hormonal Contraceptives Medroxyprogesterone	Most are fine. Conventional dosage or continuous pills for 84 days (one period every 3 months)	First-line therapy for females with dysmenorrhea or when symptoms worsen with menses. Drospirenone containing pills can have diuretic effect

Desmopressin acetate	0.1 mg at bedtime, increasing to 0.2 mg daily	Useful for those with nocturia. Monitor for hyponatremia

**Sympathetic tone modifiers**		
Beta blockers
Atenolol	12.5–25 mg daily, increase by 12.5 mg increments until optimal effect. Usual dose	First-line therapy for those with resting heart rate >100, anxiety or prominent headache. Higher doses can aggravate fatigue and lightheadedness.
Propranolol	0.5–1 mg/kg body weight	
	10–20 mg 3–4 times daily	

Pyridostigmine bromide	Rapid release: 30 mg daily, increase by 30 mg every 3–7 days to 60 mg BID or TID. Sustained-release: 180 mg daily	Effective in both POTS and neurally mediated hypotension. Can also help with GI motility problems

Clonidine	0.05 mg at bedtime. Increase after 1 week to 0.1 mg nightly. Occasionally higher doses tolerated	Consider in those with anxiety, problems with attention or insomnia. Reported to improve blood volume

**Selective serotonin reuptake inhibitor/SNRI**		
Escitalopram	5 mg daily for 2–4 weeks, increase to 10 mg daily up to max. of 40 mg daily	Consider for dysthymia, depression, or anxiety

Duloxetine	20–30 mg daily for 2 weeks, increase to max of 60–90 mg daily	Consider if myalgias are prominent

Sertraline	25–100 mg daily	

Adolescent girls with dysmenorrhea, acne or peri-menstrual exacerbation of OI symptoms can benefit from hormonal contraceptive therapy (127). Sometimes the young patient fares better when treated with continuous combined hormonal regimens that bring one menstrual period every 90 days. A long-acting injectable progesterone can be considered. The mechanism by which hormones improve OI is not entirely clear.

Improvements in symptoms and in responses to upright tilt have been reported after treatment with selective serotonin reuptake inhibitors (SSRIs) in non-depressed patients with NMH refractory to other therapies (128). In those with fibromyalgia, duloxetine (a SNRI) can be effective for pain, independent of its effect on mood. When symptoms such as anxiety, pain, dysthymia, or premenstrual syndrome are present, these medications might also be chosen.

The use of several medications with different pharmacologic effects, e.g., a vasoconstrictor, a mineralocorticoid, and a beta blocker concurrently might be necessary for the improvement of severe OI. Among those refractory to treatment, it is important to question whether the OI is exacerbated by another co-morbid condition.

Selected individuals with OI in whom medications have failed to help have occasionally been managed with weekly infusions of IV saline until symptoms stabilize. Some clinicians also utilize IV saline infusions as “rescue therapy” when orthostatic symptoms become more intense (such as after an infection). Because individuals with OI are usually hypovolemic, they can withstand a rapid infusion of 2 L of normal saline over 1–2 h. Infusions provide a more rapid restoration of intravascular volume and a larger intake of sodium than is possible orally. Peripheral IV lines are preferred, as the placement of PICC or central lines poses a risk of local infection or bacteremia (129). The efficacy of this practice has not been studied in a randomized trial and more formal study is needed.

### Joint Hypermobility, Connective Tissue Laxity, Ehler’s-Danlos Syndrome

Some 60% of young patients who meet the criteria for ME/CFS also have joint hypermobility, compared with about 20% of healthy adolescents (32). Although joint hypermobility is a physical trait that can confer advantages in athletics and dance, some patients with joint hypermobility also have constitutional symptoms including tiredness, difficulty sleeping, gastrointestinal symptoms, arthralgias, myalgias, lightheadedness, and OI suggestive of undiagnosed co-morbid ME/CFS.

Joint hypermobility is one of the clinical features of Ehlers-Danlos syndrome (EDS) (130). Other features of this heritable connective tissue disorder can include: stretchy or fragile skin, delayed wound healing, easy bruising, unusually wide or thin scars, striae in the absence of marked weight changes, blue sclerae, easy eversion of the upper eyelids, and a positive Gorlin’s sign (the ability to touch the tongue to the tip of the nose). Some with EDS develop early onset of varicose veins. In the classical form of EDS there can be hemosiderin deposition around the knees and shins. Those with the hypermobile form of EDS are more likely than those with other forms of EDS to have OI (131, 132). Constitutional symptoms that overlap with symptoms of ME/CFS are common in EDS. Fatigue and pain are substantial contributors to impaired quality of life in EDS (133, 134). The mechanism for the overlap of ME/CFS and joint hypermobility or EDS has not been explained.

Clinicians can have an increased index of suspicion for joint hypermobility if their patients have been swimmers, dancers, and gymnasts. In taking the history, the clinician should ask whether the patient has had subluxations or dislocations (including “nursemaid’s elbow” in early childhood), and whether the patient can perform “tricks” with their joints. Joint hypermobility can be overlooked unless the clinician performs specific measurements such as the Beighton score, a nine point measure in which scores of 4 or higher indicate joint hypermobility. To obtain a Beighton score, the examiner needs a goniometer to measure joint angle, assigning one point for the ability to dorsiflex each fifth finger past 90°, one point on each side for bringing the thumb to the forearm, one for more than 190° of hyperextension at each elbow, one point for more than 10° of hyperextension of each knee, and the ninth point is for the ability to place the palms on the floor bending over at the waist, with the legs straight. Some with joint hypermobility can have associated postural dysfunctions such as thoracic kyphosis, scoliosis, a head-forward posture, lumbar lordosis, and pes planus.

Diagnosing joint hypermobility is important, as the condition can be associated with pain, add to the burden of illness in ME/CFS and it requires different approaches in physical therapy. The evaluation and management of these patients often is helped by consultation with a physical therapist. For the treatment/management of pain (see “Pain”).

### Gynecological Issues

Common problems for adolescent females with ME/CFS are the exacerbation of ME/CFS symptoms (especially OI) in the week before and during the menses, and the onset of, or an increase in severity of, gynecological symptoms, including dysmenorrhea, menorrhagia, pelvic pain and premenstrual syndrome. An occasional problem is cessation of the menses following the onset of ME/CFS (see Adolescent Development). In older adolescents, sexual activity can cause post-exertional symptom exacerbation.

#### Dysmenorrhea

Clinical experience has shown that in some adolescents, the onset of dysmenorrhea may be concurrent with the onset of ME/CFS and menstrual symptoms can improve as ME/CFS symptoms improve.

#### Endometriosis

In adolescents with ME/CFS who have co-morbid endometriosis, pelvic pain and associated gastrointestinal or urinary symptoms are typically worse during the menstrual cycle. Adolescents with endometriosis are more likely than their adult counterparts to report pelvic pain that is also (or only) non-cyclical. The pain is often minimally responsive to oral contraceptive pills (OCPs) and NSAIDs. Associated symptoms can include constipation, pain with defecation, and urinary symptoms such as dysuria, urgency, frequency, and hematuria. Dyspareunia can also be present.

#### Pelvic Congestion Syndrome (PCS)

A less well recognized problem that can cause chronic pelvic pain, associated with low BP and chronic fatigue, is PCS (135–137). While this condition is more common in multiparous women, it can occasionally be present in adolescents with ME/CFS. It is associated with varicose ovarian and internal iliac veins. Symptoms include chronic, non-cyclical, pelvic pain and perineal heaviness, occasionally associated with lower back pain. Pain is usually present throughout the month, but often worsens with the menses. Unlike endometriosis, this form of pelvic pain worsens at the end of the day or with prolonged standing due to progressive distention of varicosities in the pelvis. Also in contrast to endometriosis, PCS symptoms get better with prolonged supine posture or after a night in bed. Vulvar and thigh varices are less common in adolescents with PCS than in adults.

#### Treatment

Adolescents with dysmenorrhea and/or peri-menstrual symptoms often feel better on an OCP and OI symptoms can also improve (127). The OCP can be given in monthly cycles, but for peri-menstrual symptoms, patients often do better when the pill is taken continuously for 3 months, e.g., an active pill daily for 84 days then one week of placebo pills to induce a menstrual period every 90 days. Intramuscular Depoprovera (medroxyprogesterone acetate) can also be effective. For menorrhagia, the patient should be tested for the presence of iron deficiency anemia and this should be treated if present. The menorrhagia might respond to an OCP or to cyclokapron (tranexamic acid), 1 g tid during the period. For persisting pelvic pain, consultation with a gynecologist is often helpful.

### Gastrointestinal Issues

Some young people severely affected by ME/CFS and mostly bedbound might be unable to maintain hydration and adequate nourishment orally. These patients require tube feeding (see Severely Affected and Very Severely Affected Young Patients).

Abdominal pain, nausea and reduced appetite are common in young patients with ME/CFS. Gastrointestinal conditions which can be present include: gastroesophageal reflux, gastrointestinal motility disorders, celiac disease and non-celiac gluten sensitivity, lactose intolerance, food allergies (e.g., nut, milk, eggs, and wheat), post-infectious or irritable bowel syndrome and constipation. These conditions should be considered in the differential diagnosis of gastrointestinal complaints and if present should be treated appropriately.

Another recently recognized gastrointestinal problem that can be present in up to a third of adolescents with ME/CFS is intolerance of specific food proteins (138). Milk protein is the most common offending food, but soy, wheat, and egg proteins can also cause symptoms. Many young patients are unaware that milk or other specific proteins are a problem, because immediate reactions are absent, and symptoms can be delayed for several hours after ingestion.

Symptoms that indicate the possibility of a non-IgE-mediated allergy or an intolerance of a food protein are: (a) epigastric or abdominal pain, (b) gastroesophageal reflux symptoms (heartburn, retro-sternal discomfort, acid taste in the mouth, sometimes a mucousy form of vomiting), and (c) appetite disturbance (early satiety, picky appetite) (138, 139).

Other associated symptoms can include recurrent aphthous ulcers, intermittent fevers, headaches (including migraines), worsening lightheadedness, myalgias, sinusitis, and either constipation or diarrhea. Skin testing in people with delayed gastrointestinal hypersensitivities is usually negative. Unless delayed food protein hypersensitivities are adequately addressed, they can obscure any improvements that might accompany otherwise effective treatments.

A history of suspected food intolerances should be taken. If the specific symptoms mentioned above are present and intolerance to a specific food is suspected, a trial of strict dietary elimination of the offending food for 2–4 weeks can be undertaken. Provided that the initial food that has been restricted, based on the history is the culprit, and the elimination diet is strict enough, intestinal symptoms usually begin to resolve after 10–14 days (sometimes sooner). If allergic individuals have already been restricting the offending food and then are inadvertently re-exposed, their symptoms will return. This occurrence provides support for the diagnosis. For persisting abdominal symptoms, consultation with a gastroenterologist can be helpful.

### Allergies

Allergies are more common in young patients with ME/CFS than in otherwise healthy young people and they often predate the onset of ME/CFS (140). In contrast to non-IgE mediated allergies (see Gastrointestinal Issues), IgE-mediated allergies are recognized by the presence of immediate allergic symptoms, including wheezing, pruritus, urticaria, lip and tongue swelling, and more severe features of anaphylaxis. Skin prick tests and RAST blood tests are abnormal in those with IgE-mediated allergies. A mast cell activation syndrome (MCAS) might be present.

The importance of MCAS has likely been underappreciated in the past. Symptoms can include fatigue, lightheadedness, facial flushing, rashes, itching, hives, bone and muscle pain, nausea, vomiting, abdominal pain, diarrhea, brain fog, migraines, and intolerance to multiple medications (141, 142). Joint hypermobility is also associated with a MCAS and a subset of POTS patients have MCAS (143). Some of these symptoms overlap with symptoms of ME/CFS. Recent work has identified hereditary elevations in tryptase (an enzyme released after mast cell activation) among those with POTS, joint hypermobility, and atopic disorders (144). Clinicians should have a higher index of suspicion for MCAS in those diagnosed with ME/CFS who report the symptoms listed above. Treatment of MCAS involves antihistamines and medications to stabilize the mast cell membrane such as cromolyn, quercetin and the leukotriene receptor antagonists zafirlukast and montelukast. Several detailed reviews of the clinical features, diagnostic tests and treatments of MCAS are available (141–143, 145).

### Neuroanatomic Abnormalities

Some reports have shown an overlap of symptoms between ME/CFS and anatomic abnormalities such as Chiari I malformations, congenital cervical stenosis, cervical instability, tethered spinal cord, and thoracic outlet syndrome (146, 147). Taken together, these disorders represent a very small proportion of those with ME/CFS, but their prevalence can be higher among more severely affected patients or those unresponsive to the usual interventions. Symptoms of these various abnormalities can include: sub-occipital headaches, made worse by neck flexion or extension, coughing, or straining, back and leg pain, problems swallowing, weakness, coordination difficulties, numbness in the face and limbs, and/or frequent urination. A neurological examination might reveal nystagmus, diplopia, absence of the gag reflex, hyper-reflexia and decreased sensation in the pelvis and lower limb. In those with prominent symptoms including headache, evaluation needs to exclude intracranial hypertension and intracranial hypotension. Referral to a neurologist can be helpful.

### Oral and Dental Issues

Dental and orofacial problems are found in many young people with ME/CFS, but are often neglected because the young patient is too ill to make dental office visits. Correction of dental problems can improve overall health. Commonly reported problems are xerostomia (dry mouth), dental caries, periodontal disease, bruxism, temporomandibular joint disorder (TMD) and impacted third molar teeth.

Dental visits can be stressful, result in lingering discomfort and debilitating fatigue and recovery can be prolonged. Young patients with ME/CFS and orthostatic problems might not tolerate prolonged sitting in the dental chair and some young patients have difficulty in maintaining an open mouth for the duration of dental treatment.

During dental procedures, discomfort can be minimized by the use of a mouth prop to maintain an open posture of the mouth. The smallest size mouth prop that is effective should be used. Dentists also need to be familiar with the clinical features of OI and be prepared to treat patients at risk of developing syncope. Treatment planning of elective dentistry in more extensive cases should allow time between appointments to recover from fatigue.

Dry mouth can exist on its own, result from the effects of medications or from co-morbid medical conditions. It can lead to rampant dental caries, exacerbation of periodontal disease, or oral candidiasis. Standard treatment includes increasing oral moistness with regular fluid intake, fluoride supplementation for home use, and professional dental prophylaxis.

Treatment for dental caries and periodontal disease follows standard dental practice. For those who might be concerned about whether mercury amalgam fillings should be avoided, all scientific and medical/dental organizations support the safety of amalgam.

Local anesthetics for pain relief in dental work are well-tolerated, but many young patients with ME/CFS and co-morbid OI can develop serious side effects (syncope) from the use of the epinephrine (Epi)/(adrenalin) which is included with local anesthetic agents. In these patients, a local anesthetic can be used without Epi. If a local anesthetic with added Epi is required, it should be administered with caution.

Temporomandibular joint disorders (TMD) and bruxism (tooth grinding) are more prevalent in ME/CFS patients than in the population at large and joint hypermobility co-morbid with ME/CFS is a risk factor. Bruxism often results in loss of tooth structure and can exacerbate TMD. These conditions can cause significant TM joint pain and/or headache. They should be considered in the differential diagnosis of headache in patients with ME/CFS. For bruxism, an occlusal guard can be prescribed to protect the dentition and help to alleviate TMD symptoms.

The overall approach to the management of impacted third molars (wisdom teeth) in young patients with ME/CFS is conservative. Asymptomatic impacted teeth that appear as if they will not erupt or if there is room for proper eruption, are monitored. If the teeth are partially erupted, symptomatic and/or are affecting (or will potentially affect) the existing oral/dental condition, the third molars are generally removed. The impact on the patient of tooth removal is related to the difficulty of the extraction. In those patients who would be most affected by long, difficult procedures and the possible need for IV or general anesthesia with a significant post-operative recovery time, we would recommend a more conservative approach. Each case must be evaluated individually.

## Areas of Special Clinical Concern

### Severely Affected and Very Severely Affected Young Patients

Some young patients with ME/CFS are severely ill and are wheelchair dependent, housebound, or bedridden, sometimes for months or years. Many are too debilitated to be brought to a doctor’s office. Severe post-exertional symptoms can result if a visit to a hospital emergency room should become necessary. Published data on the characteristics of this group are lacking. Our clinicians estimate that about 5–10% of patients might be severely affected and 2–5% might be very severely affected and bedridden at some time during the course of the illness, but no studies have confirmed these numbers. The course of the severe form of the illness is unpredictable. Many severely affected young patients do show varying degrees of improvement with time and some manage to return to full activities. A few remain severely affected.

Severely ill young patients are often difficult to manage and frequently have received little help from medical practitioners. They can be socially isolated and frequently have to confront disbelief in the reality of their illness from family members, school personnel, social workers and physicians. They are in need of a great deal of practical help, emotional support and comfort. In addition to medical supervision they might require support from home health services and aides perhaps overseen by a nurse manager. The patient’s caregivers might also require support. They are under great stress and can sometimes benefit from counseling.

A vital part of management is to gain the trust of the young patient and caregivers by reassuring them that the illness is recognized as a physical illness, it is taken very seriously, that the autonomy of the young patient will be respected, and that all management/treatment options that are offered will be fully discussed with the patient and caregivers and informed consent will be requested prior to implementation of any therapy. The patient can be reassured that improvement is common, even if it takes months or years, and that recovery is possible, but cannot be guaranteed.

#### Very Severely Affected Patients

At the far end of the illness spectrum are the very severely affected patients. These patients are fortunately, relatively rare. Consequently, the average practitioner can be quite unprepared when confronted by such a patient who is so extremely ill with ME/CFS. These young patients are in an exceedingly unpleasant situation. Not only are the symptoms very distressing in their own right but the patient often feels helpless and afraid and is totally dependent on her/his caretakers.

*Clinical Features of the very severely affected can include*:

- A very high degree of symptom severity.
- Bedridden with profound weakness.
- Severe body pain and hyperesthesia.
- Marked sensitivity to sound, light, touch, odors, some foods, and/or medications.
- Hyper-somnolence in the early stages.
- Severe nausea, difficulty swallowing, occasionally requiring tube feeding.
- Difficulty getting to a toilet, requiring the use of a bottle, bedpan, diapers, and/or indwelling catheter.
- Difficulty communicating their needs to a caregiver, due to speech difficulties or exhaustion.
- Severe limitations of mental activity, including short-term memory impairment.
- Severe OI, might be unable to tolerate sitting up in bed.
- Difficulty tolerating being washed in bed due to hyperesthesia.
- Emotional changes secondary to the illness: patients can be very frightened and struggle with feelings of frustration, despair, and anger.
- Vitamin D deficiency in housebound patients and prolonged bed rest can lead to osteopenia.

#### Management of the Very Severely Affected

Our advice is based on clinical experience, as there is little literature on this subject. Ideally one physician should accept responsibility for the patient’s care for the duration of the severe stage of the illness. If the patient is cared for at home, home visits are necessary. Further advice can also be given by telephone consultations or by e-mail. If the patient is very severely affected from the outset, confirmation of the diagnosis is first necessary. Where there is a marked deterioration in a moderately severely affected patient, the practitioner might need to exclude other illnesses. Consultation with physicians from other disciplines, who are familiar with ME/CFS, can be helpful. While remaining optimistic, the physician might find that she/he will need to accept the continuing severity of the illness. Likewise, the patient and the family might need to adjust their expectations to very modest levels. Although there are therapeutic options (see below), results of therapy are variable. Therefore, targets or predictions for recovery should be avoided.

The two best environments in which to care for the very severely affected patient are (a) the home and (b) an institution where long-term supportive care and symptom relief are the priorities and where staffs are familiar with the illness. Because of noise and bustle, a general hospital might be unsuitable. However, if general hospital care becomes necessary, attempts should be made to find a facility where the patient can be nursed in a very quiet location. We should caution that well-meaning attempts by hospital staff at too rapid “rehabilitation” (forcing the young patient to increase her/his activities too quickly) can lead to serious deterioration.

The best people to take care of the young patient are usually the parents. If the patient has to be admitted to an institution, attempts should be made to have one or two individual nurses be assigned to the patient.

#### Management Guidelines

- Protect the patient from undue physical, cognitive, and emotional stress.
- A very quiet environment that might include a darkened room.
- The use of patient eye pads can allow the physician to examine the patient in a low ambient light.
- Maintain fluid and nutritional intake by early recourse to tube feeding.
- General nursing care to consist of gentle help with turning, skin care and toileting, diapers might be necessary.
- Prevention of venous thrombosis by passive physiotherapy.

More detailed information on further management strategies is available (148).

#### Symptomatic Treatment

Medications should be limited to those absolutely necessary and initially prescribed in very low doses and they should be increased slowly, as tolerated.

- Provide generous pain relief (including opioids if necessary).
- Treat problems such as migraine, dysmenorrhea and orthostatic symptoms.
- Consider vitamin D supplementation in view of lack of exposure to sunlight.
- In rare cases that remain bedbound for prolonged periods, consider bisphosphonates for prevention of osteoporosis.

#### Therapeutic Options

- Immunoglobulin therapy has shown some benefit in two randomized trials (see Immune System Support). It can be given IV or IM. The IM injection can be painful.
- Regular use of IV saline can be helpful (see Orthostatic Intolerance).

#### Prognosis of Very Severe ME/CFS

There is no published literature on prognosis in very severely affected young patients with ME/CFS. One follow-up study for a period of 7–10 years, of 24 young patients classified as having severe ME/CFS, found that 7 remained severely affected, 15 improved and were no longer classified as severe and 2 patients had recovered (149).

#### Activity Management for the Homebound Patient

While cognitive behavioral therapy (CBT) and graded exercise therapy (GET) have been promoted as of value in mild and moderate ME/CFS, there is no evidence that they are of therapeutic value in very severely affected patients. Inflexible, pre-ordained GET is often harmful and leads to exacerbation of symptoms in severe cases. However, movement is important to help reduce stiffness, maintain range of motion and prevent contractures. In very severely affected patients who are confined to bed, movement is limited to tolerated activities of daily living. For those who can tolerate touch, a knowledgeable physical therapist can provide gentle, passive range-of-motion activity and gentle, passive stretching for brief periods of time (1 min at a time followed by a rest).

Any increase of activity, including moving around in bed, needs to be determined by the young patient her/himself. When possible, the gradual resumption of some activities of daily living can be encouraged, but the patient should not be pressured into this. Orthostatic symptoms might need to be treated before the patient is able to sit up for very long. Even when the patient can sit up, activity/physical therapy is usually easier when lying flat. Further progress is shown when the patient can tolerate sitting out of bed in a chair. When there is progress to the point that standing up is possible, minimal leisurely walking, for a few minutes daily can be tried. Any activity program should allow severely ill patients to pace themselves and stay within their energy envelope, however small that might be.

### The Possible Impact of ME/CFS on the Family

A child or adolescent affected with ME/CFS presents challenges for the entire family. The challenges are similar to those faced by families of children with other chronic illnesses such as leukemia or juvenile rheumatoid arthritis, but with the additional challenges of widespread disbelief in the authenticity of the illness, the pervasive social stigma associated with a diagnosis of ME/CFS and a paucity of medical professionals who are knowledgeable about the illness.

In our experience, the majority of families draw on their strengths. The parents are usually able provide the necessary care, and siblings learn to cope with diminished parental attention. When families cope well with their child’s illness, the risk of emotional damage to siblings is minimized.

Difficulties can arise when family members are ill-informed about the illness, when they do not believe that the young patient has a physical illness, when one parent needs to cease working in order to take care of the sick young person, when there is only one parent and the young person is too ill to go to school and has to be left at home alone, or when the school system is unable or unwilling to provide suitable education for the patient. As with other chronic illnesses, pre-existing marital difficulties can be compounded by the strain of dealing with a sick child. Sometimes disbelief in the authenticity of their child’s illness has led the non-custodial parent to report the custodial parent to social services as a potential case of child neglect or factitious and induced Illness/Munchausen’s syndrome by proxy. Members of the wider extended family who show disbelief in the illness can also cause problems, even from a distance.

Research has shown that siblings of young patients with ME/CFS are more anxious than normal adolescents. Siblings identified factors having a negative impact on their lives, as lack of knowledge about the illness, change in their role in the family, lack of communication within the family, new restrictions placed on family life, change of parental focus, emotional reaction to having a sick sibling, and the social stigma of being the sibling of a young person with ME/CFS. Communication within the family, social support and extra activities were found to have a positive impact (150).

Helpful strategies to support the family can include:

- Participation of both parents in the evaluation and management of the patient.
- Educating the immediate family about the illness and ensuring that siblings receive age-appropriate information.
- Enabling extended family members to also be informed about ME/CFS, with the goal of fostering their support of the nuclear family.
- Encouraging communication between family members about management of the illness.
- Having the treating physician serve as an advocate for the patient with her/his school system.

### Immunizations

Most young patients with ME/CFS tolerate standard immunizations. Immunization against human papilloma virus and hepatitis B are important for long-term health. Yearly immunization against influenza will prevent the serious relapse that can follow this illness. If possible, it should be administered when the patient is relatively well and it should be followed by 2–3 days of rest. Post immunization relapse has been reported, but is uncommon. Immunization of other family members can also help to protect the patient.

### Considerations Regarding Surgery

In general, young patients with ME/CFS requiring surgery tolerate anesthesia well. Prior discussion with the surgeon and anesthesiologist/anesthetist about this illness and issues such as OI, pain control, and possible extended recovery times should be addressed. Further recommendations are given in Appendix F.

## The School System

Myalgic encephalomyelitis/chronic fatigue syndrome is the most common cause of prolonged school absence due to illness (9, 13–16) and undiagnosed ME/CFS is also a frequent cause of poor school attendance. Absence from school is usually due to poor physical and cognitive function.

Sometimes, a knowledgeable teacher or a school nurse has been the first to suspect the illness in a student with undiagnosed ME/CFS. Some educators however, have less understanding of ME/CFS and view ME/CFS as a behavioral issue (151).

Teachers, school nurses and education officials need to be educated about ME/CFS and how it affects young people and their schooling. An educational fact sheet giving information about the disease and its impact on education is included in Appendix D.

It is helpful for school personnel to be aware of the following. The illness is very unpredictable. Symptoms vary widely between patients and wax and wane. Large fluctuations in illness severity can occur making planning and school attendance a challenge. Some students are able to attend school daily, others can only manage a part-time schedule, while others are homebound or bedbound. Early in the illness, students might be too ill to attend school and this situation can sometimes persist for months or years. Sometimes a student who is able to go school might appear fine one day and the next day they may be unable to go to school, and that inability to attend might continue for several weeks. Sometimes the student may be able to attend school in the early part of the week, but can’t manage Thursday and Friday. A common pattern of absence is that following the summer break, students with ME/CFS are able to start the school term enthusiastically, but are incapable of keeping up with the increased activity involved in maintaining their school schedule over time. They “run out of steam” and this can result in a prolonged absence from school. While this might raise suspicions of truancy, or school refusal in some school personnel, the student usually wants to attend school, but is too ill to do so.

### The Importance of Education

The physician might need to take an active role in supporting young people with ME/CFS and ensure that they receive an education that is appropriate for their physical condition. Long-term follow-up of young people with ME/CFS shows that engagement in education is a key issue that improves their ability to function regardless of whether or not they recover (23, 42). Students can become demoralized if they are asked to withdraw from school. Education helps students to fulfill their aspirations and allows important aspects of their lives such as socializing with their peer group, to develop (152). It widens the range of possible occupations in later life. Work that is low skilled is usually more physically demanding. Although students with ME/CFS have cognitive dysfunction and reduced energy reserves, intellectual reasoning is frequently retained and most students with ME/CFS are capable of keeping up with their peers in some academic classes provided that the number of their classes is strictly limited (153).

### Relationship between the School, the Student, the Family, and the Treating Physician

During regular appointments with the young patient, the physician should ask how school is going. The clinician needs to be sensitive to the relationship between the young person, her/his parents, and the school. Many families have followed a long and circuitous route to a diagnosis and the young patient can be months or years behind in school by the time a diagnosis is reached. The young patient and parents often perceive dealing with the school to be very stressful, especially so when they face disbelief about their “invisible” illness from teachers, other parents, and the young patient’s classmates (153). Compounding these stresses, parents see that their child’s academic performance has deteriorated and they might fear that she/he will not receive an education that eventually allows for employment and independent living. Many parents need to place their own lives and/or careers “on hold” in order to stay at home and take care of the young person. If both parents need to work, that can be problematic for the young patient at home alone.

Students who have understanding teachers, a flexible program, and assistance from sympathetic advocates often need less help from medical and psychological professionals. Schools differ in their response to a student with ME/CFS. Some schools are cooperative from the outset, while others resist the diagnosis, ignore the treating physician’s letters, do not follow the physician’s recommendations and might reach the point of becoming adversarial. Other schools might appear to agree with the recommendations yet never follow through with implementation. Some schools might try to make a homebound student with ME/CFS return to school too early in order to save money on services such as homebound teaching. Some educators view ME/CFS as a behavioral issue and in some instances, have instituted legal proceedings because they have wrongly believed that the cause of school absence in a student with ME/CFS was due to psychological factors such as school refusal, or the extremely rare condition of factitious disorder by proxy (Munchausen’s syndrome by proxy). As a result, young people seriously ill with ME/CFS have been removed from the care of their parents, sometimes for years.

### Impact of ME/CFS Symptoms on Learning

Several prominent ME/CFS symptoms affect the student’s ability to learn. Cognitive problems (often described as “brain fog”) are evident in most students even if they are less severely affected. Students experience mental confusion, forgetfulness, difficulty concentrating, a short attention span and a slowing of mental processing speed. Working memory can be significantly reduced and there is often increased distractibility, which can be exacerbated by noise in the classroom (85–87). IQ scores might be lower than the scores of healthy peers (154). Cognitive problems can sometimes mimic attention deficit disorder without hyperactivity. For those with more severe illness, cognitive problems are very limiting. Generally, if students are homebound, the most that they can manage are one or two essential or core subjects. Although not easy and requiring a real commitment, completing school work can give the student a real sense of achievement, which is important. The homebound student usually needs regular help from someone such as a Visiting/Homebound Teacher.

Students with ME/CFS are often unable to handle simple math calculations. They might be able to complete the steps to solve a complex problem correctly, but can make simple addition, subtraction, or multiplication mistakes. Teachers should be aware of this problem when grading tests (153).

Many young people with ME/CFS also experience orthostatic intolerance. Educators need to be aware that it is difficult for these students to stand or even sit for prolonged periods of time. These students might need to move around during lessons. They might also need access to drinks and salty snacks especially during testing. They might also be physically unable to complete long exams in one sitting.

Returning to school after a long absence can be a challenge. The illness might have occurred at a time when the student is transitioning to a new school and she/he might not be familiar with how the new school functions. The student might also have lost touch with her/his peer group. If the student needs to travel long distances to school, this is very tiring. The situation needs to be handled with understanding and patience.

### Educational Plan

Students with ME/CFS often need a personalized school schedule. The legal and procedural requirements for students to receive services for their disabilities vary significantly from place to place and are beyond the scope of this Primer.

Ideally, young people with ME/CFS will undergo an evaluation process and an educational plan should be developed by the school after consultation with the student, the student’s parents and the student’s physician. Neuropsychological testing is not routinely recommended to investigate the cognitive symptoms of ME/CFS for several reasons. First, affected ME/CFS patients often do not tolerate testing when they are at their most symptomatic, when cognitive problems might be identified. If tested on a relatively good day, they might not have scores that differ from healthy individuals. Second, neuropsychological testing in adolescents with ME/CFS is often only abnormal if the study sample is enriched with those who are reporting more difficulty with attention and memory (85, 86), or in the setting of concurrent physiological challenges such as during head-up tilt testing (50, 56). Third, this kind of testing is often not covered by insurance, and can be costly. At a practical level, the testing usually does not alter the suggested management, which most often is to decrease the volume of academic work.

Because ME/CFS symptom severity varies widely, clinicians should, whenever possible, recommend specific educational accommodations (see later) that are appropriate for the severity of symptoms. A sample physician’s letter that can be used in the evaluation process can be found in Appendix E.

Regular communication with the school is helpful. For example, a supplemental doctor’s note given to the parents can forewarn the school that a change in medication can have an adverse effect on the student’s ability to function under the provisions of his or her educational plan. When the clinician can be seen by teachers and school administrators as a partner and a valuable resource, the process of accommodating a student with ME/CFS leads to better results.

### Educational Accommodations

Any of the following can be recommended, depending on the student’s physical condition:

*In the school*:

- A person designated as a single point of contact for both teachers and the family.
- A shortened day/shortened week; the student might arrive late, leave early, attend school for only part of the day and/or only 2 or 3 days a week; students with sleep reversal might not be able to manage morning classes.
- A reduction in course load and flexible scheduling where only classes in selected subjects are attended.
- A quiet place to rest if fatigue is evident to the student or the teacher.
- Use of the elevator to access different floors.
- Exemption from or modification of, the physical education program.
- Provide homebound instruction or Distance Education for students who are partly or completely homebound.

*In the classroom*:

- Provide two sets of textbooks—one for school and one for home.
- Use the buddy system, so that someone can take notes in class, allow taping of classes, and/or give the student an outline of material taught.
- Permit the use of electronic devices such as a laptop or tablet and allow work to be completed and submitted online.
- Permit a student with orthostatic intolerance to move around during classes.
- Allow salty snacks and a water bottle for use in the classroom and especially during long tests.
- Provide tutorial or homebound instruction for work missed or if the student is too ill to attend school.

*Assessments/Testing*:

- Allow flexibility with assignments and deadlines as well as modifications of the number of problems and/or assignments to be completed.
- Significant extended time might be needed for testing as well as adjusted time of day for assessments, depending on the time when the student functions best.
- Tests/Final Exams might need to be given over several days and/or sessions with water and snacks available.

### Sports

Symptoms of ME/CFS frequently worsen after physical exertion. Some students might be able to participate in a short physical activity, but not an activity that requires stamina. The student needs to be able to recognize when she/he is experiencing the onset of fatigue and inform the teacher. When this occurs, the student *must* stop and rest. The student might want to continue, but failure to stop and rest at the onset of increased fatigue often causes a serious and prolonged relapse of symptoms. Students with ME/CFS should never be pressured to push themselves to their limits.

### Social Development

The classroom and the school lunchroom might be the only place where the student with ME/CFS is able to socialize with her/his peers. When possible, these opportunities should be facilitated (152, 153). Access to extra-curricular activities is also important for social reasons. Students who are unable to attend school often feel isolated at home, and miss their friends.

## Author Contributions

The text of this monograph was developed by consensus of all the authors, and all authors agree to the content of the manuscript. Drafts of the main chapters were revised extensively by the entire group until consensus was achieved.

## Conflict of Interest Statement

The authors declare that this monograph was written in the absence of any commercial or financial relationships that could be construed as a potential conflict of interest.

## Figures and Tables

**Figure A1:**
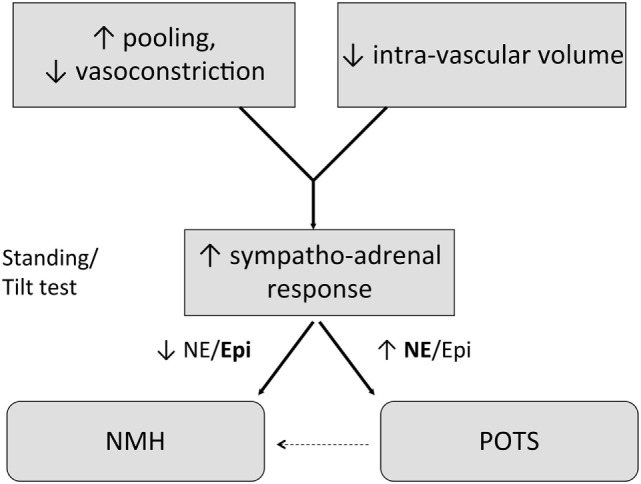
The three main pathophysiologic influences on orthostatic intolerance (OI) are shown in this Figure. Increased pooling of blood, or defective vasoconstriction in the lower half of the body, along with lower intravascular volume are each important, and can be present in the same individual. Upon assumption of an upright posture, exaggerated sympathetic nervous system and adrenal hormone responses lead to increased release of epinephrine (Epi) and norepinephrine (NE). It is postulated that the relative balance of NE to Epi is one factor in determining whether the phenotype of OI is POTS or neurally mediated hypotension (NMH). Some patients with POTS in the early phase of orthostatic testing go on to develop a classic NMH pattern later in the test.

## Appendix A

### 1994 Fukuda Research Case Definition for Chronic Fatigue Syndrome (CFS)

Patients can be classified as having CFS if they meet the following criteria:

1. The individual has had severe, clinically evaluated, persisting or relapsing fatigue for six or more consecutive months. The fatigue is not due to ongoing exertion or other medical conditions associated with fatigue. The fatigue significantly interferes with daily activities and work.
2. The individual has four or more of the following symptoms, persisting, or relapsing and concurrent with the fatigue:
  - Post-exertional malaise lasting more than 24 h
  - Unrefreshing sleep
  - Significant impairment of short-term memory or concentration
  - Muscle pain
  - Pain in the joints without swelling or redness
  - Headaches of a new type, pattern, or severity
  - Tender lymph nodes in the neck or armpit
  - A sore throat that is frequent or recurring
3. A thorough medical history, physical examination, mental status examination, and laboratory tests are necessary to identify other conditions with similar symptoms that require treatment. The diagnosis of chronic fatigue syndrome cannot be made without such an evaluation.

## Appendix B

Common conditions in the differential diagnosis of pediatric ME/CFS.

**Table:**

*Autoimmune/rheumatology* Autoimmune hepatitis Rheumatoid and other inflammatory arthritis Sjogren syndrome Systemic lupus erythematosus Wegener’s granulomatosis *Cardiovascular/autonomic* Aberrant coronary artery, coronary aneurysms Cardiomyopathy Coarctation of the aorta Patent foramen ovale Primary dysautonomia Pulmonary hypertension Rheumatic fever Valvular disease (congenital and acquired) *Endocrine/Metabolic* Addison’s disease Gonadal dysfunction syndromes Hyper- and hypo-thyroidism Hyper- and hypo-calcemia Inborn errors of metabolism Metabolic syndrome Pituitary tumors or disorders *Gastrointestinal/nutritional* Celiac Disease Chronic liver disease Eosinophilic gastroenteritis Inflammatory bowel disease Pancreatic insufficiency Vitamin D and B12 deficiency	*Hematological/immunological* Anemias Common variable immunodeficiency Leukemia/lymphoma Post-cancer fatigue *Infections* Acute mononucleosis (Epstein-Barr virus, CMV) Brucellosis Coxsackie virus Giardia and other intestinal parasites Hepatitis B or C HIV Leptospirosis Lyme and other tick-borne infections Babesia microti anaplasma phagocytophilum Ehrlichia chaffeensis *Mycoplasma pneumoniae* Parvovirus B19 Post-infectious fatigue syndrome Q fever Toxoplasmosis Tuberculosis *Neuromuscular/neuroanatomic* Cervical spine stenosis Chiari I malformation Cranio-cervical instability Tethered spinal cord Inflammatory myopathies Mitochondrial disorders Multiple Sclerosis Muscular dystrophy syndromes Myasthenia gravis Neuropathies Post-concussion syndrome	*Psychiatric* Anorexia Nervosa Bipolar disorder Chronic psychosocial stress Generalized anxiety disorder Major depressive disorder Personality disorder Post-traumatic stress disorder *Respiratory* Aspergillosis Asthma/allergies Chronic sinusitis Cystic fibrosis Sarcoidosis *Sleep disorders* Central apnea Klein-Levin syndrome Narcolepsy Obstructive apnea Periodic leg movements *Toxic substances* Alcohol or drug misuse Ciguatera poisoning Lead or mercury poisoning Organophosphate poisoning Reactions to medications, e.g., antihistamines, antipsychotics, sedatives, tricyclic antidepressants *Other conditions* Athletic overtraining or underperformance syndrome Marfan syndrome Mast cell activation syndromes

## Appendix C

### Myalgic Encephalomyelitis/Chronic Fatigue Syndrome in Children and Adolescents—Fact Sheet

Myalgic encephalomyelitis/chronic fatigue syndrome (ME/CFS) is a complex disease characterized by the body’s inability to produce sufficient energy for the normal range of human activity. There is severe, overwhelming fatigue with a substantial loss of physical and mental stamina. The patient’s energy reserves are substantially reduced. The cardinal feature is a worsening of symptoms and malaise (feeling ill) following minimal physical or mental exertion. This can persist for hours, days, or weeks and is not relieved by rest or sleep. Other symptoms include: cognitive problems (“brain fog”), unrefreshing or disturbed sleep, lightheadedness, a variety of painful conditions, and other symptoms in multiple body systems.

ME/CFS occurs worldwide. In different countries, it is labeled chronic fatigue syndrome, myalgic encephalomyelitis or the acronym, ME/CFS. A new name, systemic exertion intolerance disease (SEID) has been proposed, but is not in general use. The name “chronic fatigue syndrome” has been criticized because it can be confused with “chronic fatigue,” which is common in many other illnesses.

Myalgic encephalomyelitis/chronic fatigue syndrome usually occurs as sporadic (isolated) cases, but in 20% of patients it affects more than one family member. Clusters of cases or outbreaks of the illness (epidemics) have been found worldwide and in several of these outbreaks the illness has been prominent in schoolchildren.

#### ME/CFS in Children and Adolescents

At least one million Americans are thought to have ME/CFS and a substantial but unknown number are under the age of 18 years. Adolescents 12–17 years old are more likely than younger children to develop ME/CFS, but children as young as four years of age have developed the disease. In adolescents more girls than boys have ME/CFS.

#### Cause of the Disease

The cause of ME/CFS is unknown, but several factors may be involved. In some families, genetic factors may produce a susceptibility to the illness. Frequently the illness follows an acute infection such as infectious mononucleosis and immune system changes found in ME/CFS are similar to changes found in some viral infections. No known infectious agent has been shown to be the cause and in sporadic (non-epidemic) cases, the illness is not thought to be transmissible by casual contact. Occasionally, ME/CFS is triggered by a toxin, an immunization, or by major trauma. ME/CFS is not a psychological illness. Depression and anxiety can occur secondary to ME/CFS, as occurs in other chronic illnesses, but Major Depression and ME/CFS can be distinguished by behavioral, immunological, and hormonal testing.

#### Symptoms and Diagnosis

The diagnosis of ME/CFS is made from the characteristic pattern of symptoms and the exclusion of other fatiguing illnesses because there is no medical test for the disease. Many patients remain undiagnosed. The main diagnostic features of the illness have been incorporated into several different case definitions. We recommend the following clinical diagnostic criteria which have been found to be useful in pediatric patients:

- There is loss of mental and/or physical stamina and a substantial reduction in ability to take part in personal, educational and/or social activities.
- There is a new onset of fatigue that is not the result of ongoing exertion and is not relieved by rest. Fatigue can worsen with prolonged upright posture.
- Normal activity or mild/moderate exertion is followed by malaise (feeling ill) and worsening of ME/CFS symptoms. Recovery can take days, weeks, or months
- Sleep is unrefreshing, with disturbed quantity or rhythm that can include daytime hypersomnia, nighttime insomnia, day/night reversal.
- Any of the following cognitive problems can be present: difficulty in concentration, impaired short-term memory, difficulty understanding information and/or expressing thoughts, difficulty finding words or numbers, absent mindedness, or slowness of thought. Cognitive problems can worsen with prolonged upright posture and/or with physical or mental activity.
- Pain can be widespread or localized. Commonly seen are: chronic daily headaches, pains in the muscles, the abdomen, the joints, and/or the lymph nodes and/or sore throats. Pains can be worsened by prolonged upright posture. Rarely is pain absent.

Myalgic encephalomyelitis/chronic fatigue syndrome symptoms often fluctuate significantly in intensity during the day, or from day-to-day. To diagnose ME/CFS some or all symptoms must be present every day. The symptoms’ intensity must be mostly moderate to severe and symptoms must have persisted or recurred for at least 6 months (a provisional diagnosis and appropriate management can be instituted before 6 months). Other fatiguing illnesses must be excluded by history, physical examination, and medical testing.

Other symptoms that can be present in many, but not all, pediatric patients are:

- Orthostatic intolerance: prolonged upright posture can induce lightheadedness, increased fatigue, cognitive worsening, headaches and/or nausea.
- Hypersensitivity to light, noise, touch, odors and/or medications.
- Thermo-regulatory imbalance: low body temperature, intolerance to heat and/or cold, or cold hands and feet.
- Gastrointestinal symptoms: abdominal pain, nausea, or anorexia.

Young people with ME/CFS often do not look ill but can appear noticeably pale. Routine blood tests are frequently normal, but specialized testing can show abnormalities in patients’ immune, nervous or cardiovascular systems, or in cellular energy production.

Myalgic encephalomyelitis/chronic fatigue syndrome has often been misdiagnosed as School Refusal (school phobia), or as Munchausen’s syndrome by proxy (a condition in which a parent fabricates their child’s illness).

#### Progress and Recovery

Myalgic encephalomyelitis/chronic fatigue syndrome in adolescents usually starts suddenly with a fever and flu-like symptoms. Sometimes the onset is gradual. In younger children, a gradual onset over months or years is more likely. It can be difficult to diagnose ME/CFS in younger children because they may not recognize that their fatigue and other symptoms are abnormal. The diagnosis can be made retrospectively when the child is older. The first sign of the illness might be the child’s marked limitation of activity, noticed by a parent or teacher. Young people with ME/CFS can be very ill at the onset of the illness, but because routine blood tests are frequently normal, the diagnosis is often uncertain. Even when no other illness is found, a definitive diagnosis of ME/CFS cannot be made for 6 months, but a provisional diagnosis can be made sooner. Early diagnosis can lessen the impact of the illness by ensuring an appropriate management plan.

The severity of ME/CFS varies. Some young patients are severely disabled and bedridden, while others can go to school and a few can even do sports. Most are between these extremes. Over time, slow improvement is likely. Recovery rates are uncertain but rates of up to 40% have been reported. Dramatic improvement is more likely to occur in the first four years. Remissions and relapses are common. Relapses can be caused by overexertion or by infectious illnesses. Young patients whose health improves to near pre-illness levels are likely to find that they need more rest than their contemporaries.

#### Management/Treatment

Establishing the diagnosis of ME/CFS can relieve uncertainty in the minds of the patient and the parents. There is currently no medication or intervention that will cure the disease. Management differs between individuals. Determining the optimum balance of rest and activity (pacing of activities) can help prevent post-exertional worsening of symptoms. Medications are helpful to treat pain, insomnia, and orthostatic intolerance. Young patients with ME/CFS commonly respond to lower dosages of many medications. Advice on nutrition can be helpful. Supportive psychotherapy can sometimes benefit mildly affected young patients, but inflexible, graded exercise (GET) is harmful and can lead to worsening of symptoms.

#### Education

Myalgic encephalomyelitis/chronic fatigue syndrome is the most common medical cause of long-term absence from school. Most students with ME/CFS fall behind in their education. Students with ME/CFS might need a personalized school schedule and the school might need to provide reasonable accommodations and/or home tutoring. Legal and procedural requirements for students to receive services for their disabilities vary significantly from place to place.

## Appendix D

### Myalgic Encephalomyelitis/Chronic Fatigue Syndrome: Fact Sheet for Schools

Myalgic encephalomyelitis/chronic fatigue syndrome (ME/CFS) is characterized by the body’s inability to produce sufficient energy for the normal range of human activity and the patient’s energy reserves are substantially reduced. The main symptoms include:

- Severe, overwhelming fatigue with loss of mental and/or physical stamina and a substantial reduction in ability to take part in personal, educational and/or social activities.
- The cardinal symptom of worsening of symptoms and malaise (feeling ill) following minimal physical or mental exertion which can persist for hours, days, or weeks and is not relieved by rest
- Cognitive problems (“brain fog”), unrefreshing or disturbed sleep and a variety of painful conditions (rarely pain is absent). Cognitive problems may worsen with prolonged upright posture.

There is no medical test for the illness. The diagnosis is made from (a) the characteristic pattern of symptoms (b) some or all of the above symptoms must be present every day for at least 6 months, (c) symptoms must be moderate or severe, and (d) other fatiguing illnesses must be ruled out by history, physical exam, and medical testing

Additional symptoms are often present and include:

- Orthostatic intolerance, which is the development of symptoms due to prolonged upright posture (standing or sitting) that result in lightheadedness (and sometimes passing out), increased fatigue, cognitive worsening, headaches, and/or nausea.
- Hypersensitivities to light, noise, touch, odors, and/or medications.
- Problems with thermoregulation such as low body temperature, intolerance to heat and cold and/or cold hands, and feet.
- Gastrointestinal symptoms such as abdominal pain, nausea, and/or loss of appetite.

ME/CFS often starts suddenly following a viral illness, but it can start gradually. Symptoms can vary unpredictably in severity from day-to-day and from week-to-week. Students with ME/CFS often do not look ill, but can appear to be very pale. The cause of ME/CFS is unknown, but the disease is not thought to be transmitted by casual contact. There is currently no medication or intervention that will cure ME/CFS. Successful management is directed toward determining the optimum balance of rest and activity to help prevent post-exertional worsening of symptoms. Medications are helpful to treat pain, insomnia, orthostatic intolerance and other symptoms

#### Educational Implications

Myalgic encephalomyelitis/chronic fatigue syndrome is the most common cause of long-term absence from school due to illness. Absence from school is usually due to poor physical and cognitive function, not behavioral factors. Some students can attend school daily, others can manage part-time, while others are homebound and some are confined to bed. Sometimes a student has enough energy for school at the start of the week, but is unable to manage school on Thursday and Friday. ME/CFS is unpredictable. A student might appear fine one day but the next day might be unable to come to school, sometimes for several weeks. There might also be long periods where she/he is unable to complete schoolwork at home.

Most students with ME/CFS experience worsening of their school performance as ME/CFS symptoms impact education. Cognitive problems include confusion, difficulty with concentration, slow information processing, short-term memory problems, impaired word retrieval, and easy distractibility. These problems can manifest in several ways. If the teacher has asked the student to complete a task and she/he is interrupted, she/he might not remember what the teacher asked her/him to do. The student might require extra time to answer questions or complete assignments. The student might temporarily lose the ability to retrieve information learned the day before. Classroom noise can worsen distractibility. This distractibility can result in teachers perceiving that the student is uninterested, or cannot pay attention. Students with ME/CFS may be unable to handle simple math calculations. They can often complete the steps to solve a complex calculus problem, but make simple addition or multiplication mistakes. Intellectual reasoning is usually retained in spite of cognitive problems, and many students are capable of taking academic classes with their peers, provided that that the number of their classes is strictly limited.

Symptoms of ME/CFS worsen after physical exertion. Participation in sports can deplete the student’s energy reserves. Some students may manage a short physical activity, but not an activity that requires stamina. The student needs to be able to recognize when she/he is experiencing the onset of fatigue, inform the teacher and *must* stop and rest. The student might want to participate, but failure to stop and rest at the onset of increased fatigue can cause a serious relapse of symptoms. Students with ME/CFS should never be pressured to push themselves to their limits.

#### Educational Accommodations

Students may need a personalized school schedule. The following accommodations can be helpful:

*In the school*:

- A single point of contact for teachers.
- A shortened day/week, the student might need to come in late, leave early, and/or attend school for only 2 or 3 days a week.
- A reduction in course load and flexible scheduling where only classes in selected subjects are attended.
- A quiet place for the student to rest if fatigue is evident to the student or the teacher.
- Use of the elevator to access different floors.
- Exemption from, or modification of, the physical education program.
- Provide homebound instruction or “Distance Education” for students who are partly or completely homebound.

*In the classroom*:

- Provide two sets of textbooks—one for school and one for home.
- Give clear directions with frequent feedback.
- Help the student to organize work with the use of an assignment book and/or online calendar to record assignments and work completed.
- Use the buddy system, so that someone can take notes in class, allow taping of classes, and/or give the student an outline of material taught.
- Use multi-sensory instruction, e.g., visual aids like graphic organizers and non-linguistic representations to better suit the learning style of the student.
- Teach tasks serially instead of having the student multitask, break work down into manageable segments—short frequent projects are better than long-term projects.
- Permit the use of electronic devices such as a laptop or tablet and allow work to be completed online.
- Permit a student with orthostatic intolerance to move around during classes and allow salty snacks and a water bottle in the classroom.
- Provide tutorial or homebound instruction, if the student is too sick to attend school.
- Allow flexibility with assignments and deadlines, and modifications of the number of problems and/or assignments to be completed.

*Assessments/testing*

- Test knowledge of material in multiple ways, e.g., oral instead of written tests, or course work instead of exams.
- Extended time and a quiet room might be needed for testing as well as adjusted time for assessments depending on the time of day when the student functions best.
- Tests/Final Exams may need to be given over several days or sessions, with water and snacks available.

*Curriculum*

- Identification/prioritization of essential content.
- Focus on mastery of skills rather than completion of assignments.
- Use of frequent short-term projects instead of long-term projects.
- A reduction in the number and length of assignments, projects, quizzes, and tests.

#### Social Development

The classroom and the school cafeteria might be the only place where the student with ME/CFS is able to socialize with her/his peers. When possible, this should be facilitated. Access to extra-curricular activities is also important for social reasons.

#### Resources

National Association of Special Education Teachers Chronic Fatigue Syndrome, www.naset.org/3349.0.html. Association of young people with ME, AYME www.ayme.org.uk. Association of New Zealand Myalgic Encephalopathy Society, www.azmes.org.nz.

## Appendix E

### Sample Physician’s Letter

This letter is an example and it needs to be adapted so that it is applicable to the student’s country and is very specific for the student’s particular symptoms.

Dear Special Education Administrator:

Or Special educational needs co-ordinating officer:

Or School Principal:

Jane XXXX is under my care for myalgic encephalomyelitis/chronic fatigue syndrome (ME/CFS). This illness has caused Jane to experience severe fatigue and a lack of physical and mental stamina. Jane’s body is unable to produce sufficient energy for the normal range of activities for her age and her energy reserves are substantially reduced. If Jane exceeds her diminished energy reserves, it can take her several days, weeks, or even months to recover.

The illness has caused Jane to have cognitive problems, muscle pains, poor sleep and increased sensitivity to light, to noise and to a cold environment. Jane also tends to feel faint if she has to stand up for any length of time. Jane’s cognitive problems can cause her to have difficulty concentrating, understanding complex concepts, remembering words, working with numbers, a poor working memory and increased distractibility. Classroom noise can worsen these difficulties. It is important to note that intelligence is not compromised by this illness and provided that Jane has appropriate accommodations, she can be expected to attain educational success.

The occurrence and severity of Jane’s symptoms are quite unpredictable and vary from day-to-day. The illness may result in Jane missing a significant numbers of days during the school year, or she might need to arrive late or leave early from school. Jane will only be able to manage a reduced subject load. It will therefore be necessary for the school to be accommodating and flexible in response to Jane’s likely absences and reduced curriculum.

Currently, Jane is not well enough to engage in any sports. Please excuse her from physical education activities until I am able to confirm if and when her health has improved enough that these activities will not make her illness worse.

I support an application for educational services appropriate to Jane’s needs. A list of my recommendations for accommodations and modifications for Jane is attached. Please let me know if I can be of further assistance.

Sincerely,

XXXXXXXXXXXXX, MD

## Appendix F

### Pediatric Patients with ME/CFS Anticipating Surgery: Special Considerations*

Myalgic encephalomyelitis/chronic fatigue syndrome is a complex, debilitating disease characterized by severe overwhelming fatigue with a substantial loss of physical and mental functional capacity. The cardinal feature is malaise and worsening of symptoms following minimal physical or mental exertion which can persist for hours, days, or weeks and is not relieved by rest or sleep. Other symptoms include cognitive impairment, non-restorative sleep, generalized, or localized pain, and immune, neurological and/or autonomic symptoms. Remissions and relapses are common.

Most young people with ME/CFS demonstrate vasovagal syncope, postural tachycardia syndrome (POTS), neurally mediated hypotension (NMH) and increased venous pooling on tilt table testing. Syncope can be precipitated by catecholamines (epinephrine), sympathomimetics (isoproterenol), and vasodilators (nitric oxide, nitroglycerin, α-blockers, and hypotensive agents). Care should be taken to hydrate patients prior to surgery and avoid drugs that stimulate neurogenic syncope or lower BP. Many patients benefit from extra IV fluids in the perioperative period.

Propofol, midazolam, and fentanyl are generally well-tolerated. However, studies in adults have shown that some patients with ME/CFS can be sensitive to histamine-releasing anesthetic agents (such as pentothal) and muscle relaxants (curare, Tracrium, and Mevacurium). If possible, they are best avoided. Young patients can also be extremely sensitive to sedative medications—including benzodiazepines and antihistamines.

Herbs and complementary and alternative therapies are often used by patients with ME/CFS. The anesthesiologist/anesthetist should ask patients about such therapies (155). Of most concern are:

- Garlic, gingko, and ginseng—can increase bleeding by inhibiting platelet aggregation.
- Ephedra (ma huang)—can cause hemodynamic instability, hypertension, tachycardia, arrhythmia.
- Kava and valerian (increase sedation).
- Echinacea—allergic reactions and possible immunosuppression with long-term use.
- St. John’s Wort—multiple pharmacological interactions due to induction of Cytochrome P450 enzymes.

The American Society of Anesthesiologists recommends that all herbal medications be discontinued 2–3 weeks before an elective procedure. Stopping kava (also known as awa, kawa, and intoxicating pepper) can trigger withdrawal, so this herbal should be tapered over 2–3 days.

Although early studies have shown mild HPA depression in some adolescents with ME/CFS (62), supplemental cortisol is seldom needed unless the patient is severely ill. Those patients who are being supplemented with cortisol should have their doses doubled or tripled before and after surgery.

Mild immunological abnormalities can be found in this illness, but patients are not immunocompromised and are, no more susceptible to opportunistic infections than the general population.

#### Summary Recommendations

- Ask about herbs and supplements, and advise patients to taper them off at least 1 week before surgery.
- Hydrate the patient prior to surgery. Consider additional post-operative fluids.
- Use hypotensive agents, sympathomimetics, catecholamines, and vasodilators with caution.
- Use sedating drugs sparingly.

*Based upon “Considerations Prior To Surgery for Adult ME/CFS Patients” by Dr. CW Lapp MD, Director, Hunter-Hopkins Center.

## Appendix G

### Orthostatic Intolerance

Individuals with OI develop an exacerbation of symptoms with quiet upright posture and the amelioration (although not necessarily complete resolution) of those symptoms with recumbency (156). OI can be associated with a variety of treatable circulatory disorders, the most commonly recognized of which in pediatric ME/CFS are postural tachycardia syndrome (POTS) and NMH (48–55, 157).

The main symptoms of chronic OI are shown in Table A1. These clinical features usually are attributed to two prominent physiological changes in response to upright posture: 1. a reduction in cerebral blood flow, and 2. an exaggerated adrenergic compensatory response to upright posture (156). As can be readily appreciated, the symptoms in Table A1 overlap with many of the features of ME/CFS, including fatigue, exercise intolerance, cognitive problems, and headaches. In all controlled studies, compared to healthy individuals, those with pediatric ME/CFS have a higher prevalence of abnormal circulatory responses to upright tilt testing or prolonged standing (48–55). Studies that employ measures of HR variability consistently demonstrate a sympathetic predominance of HR variability in pediatric ME/CFS (or a relative loss of parasympathetic activity) (158, 159), along with elevated levels of supine catecholamines compared with healthy adolescents (160). The relevance of OI syndromes for ME/CFS extends to the fact that open (unblinded) treatment of the circulatory abnormalities is associated with improvement in ME/CFS symptoms and function in a large proportion of adolescents (48, 51, 157, 161).

**Table A1:** Symptoms of orthostatic intolerance (in approximate order of frequency of occurrence).

Fatigue Intolerance of low impact exercise Lightheadedness Near-syncope or syncope	Blurring of vision Headaches Problems with short-term memory and concentration nausea	Chest pain Palpitations Diaphoresis Tremulousness Vomiting

### Physiologic Responses to Upright Posture

In response to standing, approximately 500–750 mL of an adult’s blood volume is redistributed to vessels below the level of the heart. A similar circulatory change is thought to occur in adolescents. At all ages, the response to the gravitational pooling of blood is a reduction in blood return to the heart, in turn leading to a drop in cardiac output, less stretch of baroreceptors, and ultimately less blood flow to the brain. The nervous system response involves an increase in sympathetic neural outflow, improved vasoconstriction, up to a 40 bpm increase in HR, and the return of enough venous blood to maintain BP and cerebral perfusion (162–164). Symptoms of OI can appear when these adjustments do not occur in an efficient manner.

Figure A1 illustrates the three main physiological contributors to OI: excessive dependent pooling of blood, low blood volume, and an exaggerated catecholamine response. A variety of pathophysiologic influences can contribute to increased peripheral pooling of blood or decreased vasoconstriction. Those with ME/CFS often have low blood volume (57–59), which has been associated with lower renin:aldosterone ratios (58) and with lower ADH levels (165). Physical inactivity results in reductions in plasma volume, thereby aggravating symptoms of OI and interfering further with daily function (102, 166, 167). In patients with increased pooling of blood in the peripheral circulation or low blood volume (or both), the assumption of upright posture is associated with less return of blood to the heart, and a markedly increased catecholamine response (168, 169). Goldstein has suggested that the relative balance of Epi to NE can influence the pattern of circulatory response (170). ME/CFS patients with more NE can maintain BP longer because NE has vasoconstrictor properties, while its effects on the heart cause tachycardia. In contrast, Epi causes skeletal muscle vasodilation, and can therefore contribute more to hypotension. Patients who develop tachycardia early during tilt table testing can develop NMH as the orthostatic stress becomes more prolonged, usually 5–59 min after the initial orthostatic tachycardia (171–173). As described in this Primer, the treatments of these disorders overlap almost completely.

### Forms of OI in ME/CFS

#### Orthostatic Hypotension

Orthostatic hypotension is defined by a sustained BP reduction of at least 20 mm Hg systolic or 10 mm Hg diastolic during the first 3 min after assuming an upright posture (124). This problem is rarely seen in children except at times of febrile illness, acute dehydration, hemorrhage, adrenal insufficiency, excessive histamine release, or as a response to certain medications.

A more common pediatric variant, termed *initial orthostatic hypotension* (IOH), is characterized by a transient drop in BP immediately after standing, but resolving within 60 s. Its recognition requires a continuous beat-to-beat BP measurement device. The diagnosis is missed by standard, automated sphygmomanometer measurements (174, 175). Although this is not usually a condition that requires clinical treatment, chronic orthostatic symptoms (e.g., LH) in those with IOH have been reported (54), suggesting that they can develop other orthostatic abnormalities (such as POTS or NMH) on more prolonged monitoring.

#### Postural Tachycardia Syndrome (POTS)

Postural tachycardia syndrome is increasingly being recognized as the most common form of OI in pediatric ME/CFS. As is the case for pediatric ME/CFS, postural tachycardia syndrome is more common in females than males, is more common after the onset of puberty, and often follows an apparent infectious illness (124, 156, 176–178).

POTS should only be diagnosed (a) if there is no OH in the first 3 min of standing or tilt testing, (b) if there is an increase in HR of ≥40 beats per minute (bpm) from recumbent to upright in the first 10 min of standing or passive upright tilt, or if there is a HR >120 bpm during the first 10 min upright (124). The adult HR criterion is lower, a 30 bpm change; using the adult HR definition would mis-classify approximately 40% of healthy adolescents as having POTS (179). Therefore, a 40 bpm increase in HR is necessary as the criterion for adolescents.

Regardless of age, the diagnosis of POTS also requires that (c) the HR changes must be accompanied by the provocation of typical orthostatic symptoms. POTS should not be diagnosed by a HR elevation alone, if the individual is dehydrated, or is being treated with vasodilating medications. Conversely, the diagnosis can be obscured if the individual is tested after consumption of substances that increase plasma volume, such as salt tablets, or if the patient is being treated with serotonin reuptake inhibitors, or stimulant medications. All these can be used to treat OI.

#### Neurally Mediated Hypotension

Neurally mediated hypotension was initially identified in pediatric and adult ME/CFS in the mid-1990s (48, 49, 51). Reported frequencies of POTS were substantially lower in the early reports. For reasons that are unclear, POTS is now recognized more frequently, possibly related to increased dietary sodium intake in ME/CFS subjects over time, to the duration of orthostatic stress, or to other as yet unidentified factors.

Reflex forms of hypotension and syncope include vasovagal syncope, neurocardiogenic syncope, delayed OH, and NMH (180). The latter term is the most accurate description of the events seen in ME/CFS patients who have chronic daily orthostatic symptoms in the absence of clinical syncope and develop a characteristic drop in BP during tilt testing.

Neurally mediated hypotension in both children and adults is defined as at least a 25 mm Hg reduction in systolic BP with a slowing of the HR at the time of pre-syncope or hypotension, identified during standing or upright tilt table testing, and associated with provocation of characteristic orthostatic symptoms (48). It occurs after the 3 min cutoff for OH. HR can be elevated early in the test, but slows when hypotension becomes severe. Like ME/CFS, NMH is more common in females (180). Hypotension can be missed if the period of orthostatic testing lasts less than 10 min; the diagnosis usually requires prolonged head-up tilt testing for 40–60 min (51).

The pathophysiology of NMH is reviewed elsewhere (163, 180–184), but as in POTS, in NMH the reduction in venous return initiates a series of responses that ultimately results in withdrawal of sympathetic tone and relatively unopposed vagal tone. Sympathetic withdrawal in turn leads to vasodilation, and if the individual remains upright, the ultimate result can be either a profound drop in BP or syncope, occasionally associated with a junctional bradycardia or brief periods of asystole. Marked elevations in Epi levels are present in the period before syncope (169, 184). Factors that can provoke NMH include prolonged standing, heat, sudden stretch of mechanoreceptors in the gastrointestinal tract, bladder, and lungs, the sight of blood, and situations involving pain (180). Often a combination of precipitating factors is present.

Although many subjects with NMH only have isolated episodes of syncope separated by long periods of normal function, chronic daily orthostatic symptoms can also be present. Chronic fatigue as well as impaired quality of life is reported in those referred for evaluation of recurrent syncope (185–187). Approximately 25% of patients evaluated for chronic OI by some groups have had neurocardiogenic syncope without POTS (170). Thus, not all chronic OI is related to POTS physiology. More work is needed to determine the mechanisms by which cognitive symptoms persist despite adequate control of HR and BP.

While there is variability in the rates of OI reported in adult studies of ME/CFS, provocation of fatigue with orthostatic stress is almost universal at all ages among those with ME/CFS and in the related condition fibromyalgia (50, 188). In pediatric ME/CFS, rates of OI exceed 90% of affected subjects (157, 189) and differences in cardiovascular responses can be detected with as little as 20° of upright stress (55).

Individuals who experience chronic symptoms of OI but do not meet criteria for POTS or NMH on orthostatic testing can be classified as having low orthostatic tolerance. Treatment can be offered in the same manner as it would be for those with confirmed circulatory disorders, provided symptoms are sufficiently severe.

### Testing for OI

Two main forms of testing for OI—standing tests and head-up tilt tests—are used in clinical practice. Methods for conducting each test vary. There is no gold standard test for OI, and we do not know all of the pathophysiological contributors to symptoms such as lightheadedness, as some classical “orthostatic” symptoms can be reproduced by maneuvers that do not involve upright posture.

#### Head-Up Tilt Table Testing

Head-up tilt table testing this is usually performed with a motorized table with a foot board for weight-bearing (190). The patient lies supine, loosely restrained by safety straps to prevent injury if loss of consciousness occurs. After a variable period of supine rest, usually 15 min but in some studies up to 60 min, the tilt table is brought upright, usually to 60–70 °. Higher angles for tilt testing can produce syncope in healthy controls. The duration of upright tilt also varies between centers, depending on which form of OI is being evaluated. Tilt testing for 10 min is sufficient to detect POTS, but upright tilt for 45–60 min is required for detection of NMH. A variety of methodological factors can affect tilt response (190–192), including the degree of movement permitted, the ambient temperature, the duration of the pre-tilt fast, whether the patient is permitted to remain on medications, the pre-test sodium intake, instrumentation (arterial catheters are associated with higher rates of syncope), pharmacological agents used to provoke hypotension [e.g., isoproterenol (193), nitroglycerine], and the definition of an abnormal test (192). We do not endorse reproduction of syncope as the end point of testing, as POTS and NMH can be identified and the table returned to the horizontal position before syncope occurs. In most laboratories, HR is monitored continuously, as is BP, although intermittent BP measurements are performed in some centers, approximately every 1–2 min for 5–10 min, then every 5 min unless pre-syncopal symptoms are identified. Some laboratories measure end-tidal CO2 to detect orthostatic hypocapnia (194–196). Before and during the test, at approximately 5 min intervals, the patient is asked to rate changes in symptoms on a 0–10 scale (lightheadedness, warmth, fatigue, headache, shortness of breath, mental fogginess, and pain).

Not all individuals with ME/CFS and orthostatic symptoms require head-up tilt testing. For example, if the clinical history is consistent with neurocardiogenic syncope, head-up tilt testing is no longer thought to be necessary for the diagnosis (191, 192, 197). We would caution that care must be taken in the most impaired individuals with ME/CFS given the potential for an increase in symptoms during and after the tilt test. In this group, brief orthostatic supine, seated, and standing vital signs over 1–3 min in some instances are sufficient as an indication of orthostatic tolerance. In all individuals with ME/CFS who develop a provocation of their typical symptoms during the tilt test, we recommend administration of 1–2 L of normal saline as a means of improving autonomic tone and avoiding a prolonged post-tilt exacerbation in ME/CFS symptoms (172).

#### Standing Tests

Two forms of standing tests are described in the ME/CFS literature: active standing (59, 60) and leaning against a wall (131). Either form of standing test can be performed in a medical office. The test does not require specialist consultation or specialized equipment, and is much less costly than a tilt table test. The duration of supine rest before the test varies, but a consistent baseline HR and BP in adolescents can be obtained within 5–15 min. Although the duration of upright posture can be as long as 60 min (59), most centers perform a 10-min standing test to identify POTS and to ascertain for worsening of ME/CFS symptoms during orthostasis, acknowledging that such a brief period of testing will miss many instances of NMH. As in the head-up tilt test, symptoms are recorded immediately before standing and at 1–2 min intervals when upright. The test must be witnessed to avoid the potential for injury in the event of syncope. Movements such as fidgeting and tensing the lower limb muscles are discouraged. See OI: Standing Test protocol and Standing Test Data Sheet.

Few papers have compared the various orthostatic testing methods. When tilt testing and active standing have been compared, this has usually been just for brief periods of upright posture (167, 198). During the first 5 min upright, tilt and active standing generate similar HR changes (167, 198, 199). Beyond the first 5 min, however, Plash and colleagues recently demonstrated that passive tilt testing provokes a larger HR change in those with POTS than does active standing (199). Application of lower body negative pressure is a method of orthostatic testing, but it is rarely performed outside of research settings (160).

### OI: Standing Test Protocol

The standing test begins with the subject lying supine with an automated BP cuff set to measure the baseline HR and BP. These are recorded each minute for 5 min. At the fifth minute, the patient’s current symptom intensity is rated on a scale of 0–10.

The patient is then instructed to stand, leaning back against a wall in a comfortable but motionless position. The patient’s feet should be 2–6 inches away from the wall. The HR and BP are recorded and any symptoms are noted each minute for a maximum of 10 min. At the conclusion of the standing period the patient is instructed to lie supine again, while the BP, HR, and symptom intensity are measured for a further 2 min.

Specific instructions for the patient are as follows:

“We’d like you to stand as still as possible for up to 10 minutes. During the standing test you must be as motionless as possible in order to get an accurate result. Therefore, try not to wiggle your toes or fingers, scratch your nose, or move your arms or legs. We will monitor for any movements and will remind you not to move or wiggle. We want you to tell us if you are feeling anything different or uncomfortable during the test. Be as specific as possible. We need to know if you feel you can’t stay standing any longer, and if this is the case you can sit down. It is not necessary to remain standing for the entire 10 minutes, but we’d like to measure how long you can do this. Each minute we will check your blood pressure and heart rate with an automatic blood pressure measuring device.”

### Comments

If the subject reports any symptoms, list these in the comments column of the data form, corresponding to the time recorded for the BP and HR. The comment section should also note if and when the subject had to sit down and mention whether the BP and HR were performed sitting or while still standing.

### Definitions

Postural tachycardia syndrome (POTS): an increase in HR of at least 30 beats per minute (bpm) in adults or 40 bpm in adolescents from the supine baseline to the maximum HR during the 10 min of standing, together with the reproduction of orthostatic symptoms. Florid POTS is considered any HR >120 bpm.

Orthostatic hypotension: a reduction in systolic BP of >20 mm Hg or in diastolic BP >10 mm Hg in the first 3 min.

Neurally mediated hypotension: at least a 25 mm Hg decrease in systolic BP from baseline supine values, with no associated increase in HR, together with the emergence of pre-syncopal symptoms.

### OI Standing Test Data Sheet

Name: ______________________Date of Test: ___________

Medications in last 2 weeks: ____________________________

**Table:**

	Heart Rate	Blood Pressure	Symptoms, Comments
**Supine**			
1 min			

2 min			

3 min			

4 min			

5 min			

**Standing**			
1 min			

2 min			

3 min			

4 min			

5 min			

6 min			

7 min			

8 min			

9 min			

10 min			

**Supine**			
1 min			

2 min			
